# Supplementary material for: Variation in proviral content among human genomes mediated by LTR recombination
Source: Mob DNA. 2018 Dec 18;9:36. doi: 10.1186/s13100-018-0142-3 (PMC6298018; doi:10.1186/s13100-018-0142-3)
Supplement: Supplementary file 1 — IGV screenshot of the dimorphic HERV-W locus 18q21.1_W2. (PDF 30 kb) [file 13100_2018_142_MOESM1_ESM.pdf]

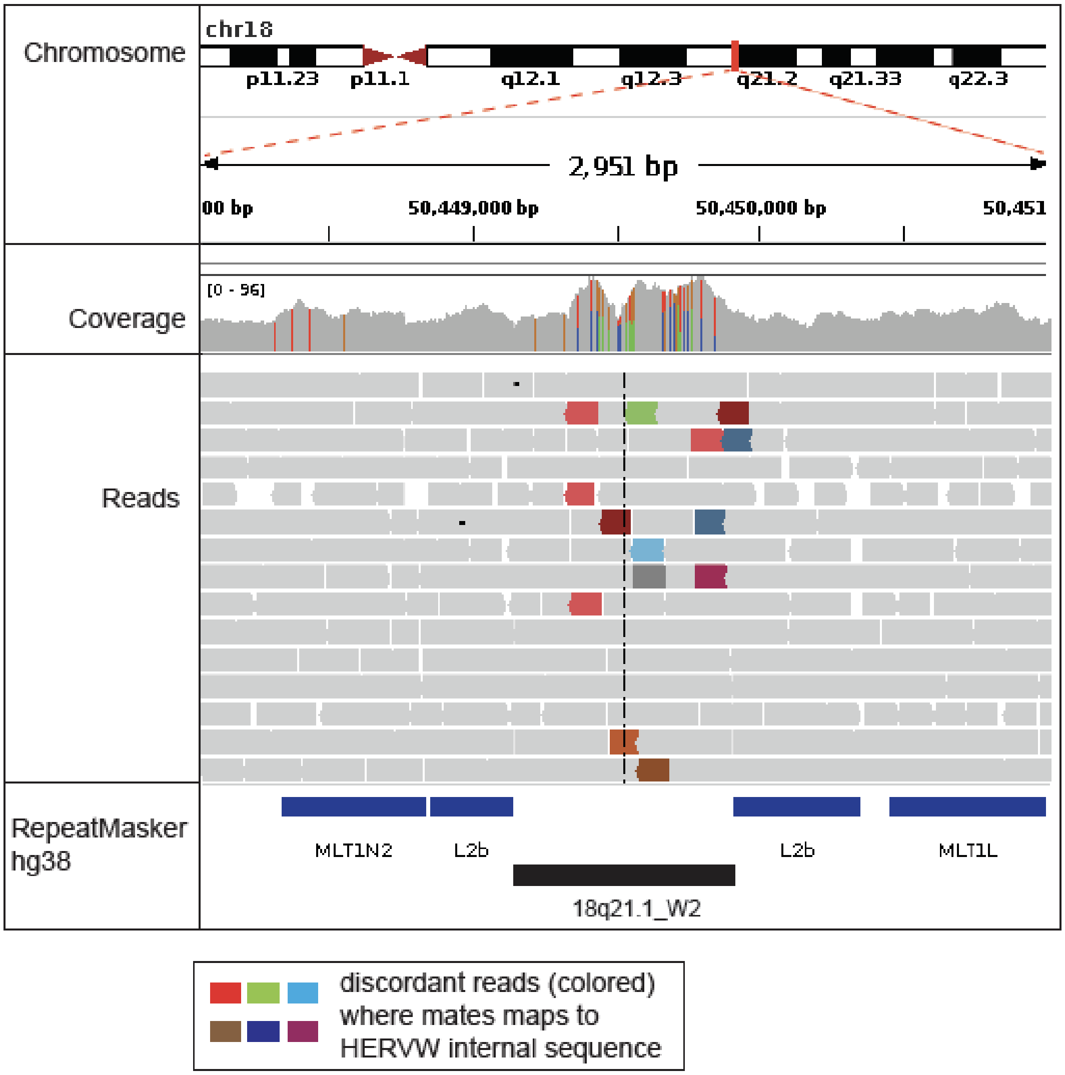

Additional file 1. IGV screenshot of the dimorphic HERV-W locus 18q21.1_W2. The top panel shows the position on the chromosome and hg38 coordinates. The Coverage track shows the number of reads that covers each base at the locus. The numbers within brackets [0-96] shows the range of reads covering the visualized region. The Reads track shows a sample of the reads mapped to that locus. The colored reads are discordant reads whose mates have unexpected insert size or inconsistent orientation. The RepeatMasker track shows the position of transposable elements and repeats identified in that chromosomal interval. The presence of discordant reads with mates mapping to internal HERV-W coding sequence is used to infer the presence of provirus at the locus. The increase in read coverage pertaining to the solo LTR region is also indicative of presence of provirus, as the reads from 2 LTRs of a provirus allele map to the solo LTR. The candidate is experimentally verified to be homozygous for provirus at that locus.
