# Supplementary material for: Variation in proviral content among human genomes mediated by LTR recombination
Source: Mob DNA. 2018 Dec 18;9:36. doi: 10.1186/s13100-018-0142-3 (PMC6298018; doi:10.1186/s13100-018-0142-3)
Supplement: Supplementary file 2 — HERV candidates identified as solo LTR to provirus variants using findprovirus pipeline. (PDF 96 kb) [file 13100_2018_142_MOESM2_ESM.pdf]

Additional file 2. HERV candidates identified as solo LTR to provirus variants using *findprovirus* pipeline

HERV name	Hg38 coordinates	discordant reads	Mappability scores*	#individuals	NCBI accessions	Previously reported
1p31.1_K2	chr1:73129298-73130265	1-16	0.143-0.256 (0.222)	264	AC212296.2 AC244103.1	(46)
5q11.2_K3	chr5:59463786-59464754	1-6	0.229 (0.229)	164		This study
K111/K105	chrUn_GL000219v1:175210-176178	1-35	NA	NA	GU476554.2	(29,44)
12q13.2_K1[$]	chr12:55333531-55334299	-	(0.053)	-	-	(39)
10p12.1_K2[$]	chr10:26893570-26894337	-	(0.024)	-	-	(38)
4q22.1_H8	chr4:91045790-91046151	1-23	0.276-1.0 (0.521)	265	AC226758.3 AC210774.3	This study (PCR verified)
5p15.31_H2	chr5:7262337-7262742	1-6	1.0 (1.0)	117	AC226159.3	This study (PCR verified)
18q21.1_W2	chr18:50449151-50449914	3-33	0.413-1.0 (0.707)	194	AC241669.2 AC203657.2 AC241258.1 AC013759.6	This study (PCR verified)

Footnotes: NA- Not applicable. (*) range of mappability scores identified for the genomic regions where discordant reads are mapped. The average mappability is given in brackets. These data are collected from the individual that had the most informative discordant reads (where mates had significant homology with the respective HERV internal sequence) mapped to the solo LTR. ($) Not identified as dimorphic using our pipeline.
