# Supplementary material for: Variation in proviral content among human genomes mediated by LTR recombination
Source: Mob DNA. 2018 Dec 18;9:36. doi: 10.1186/s13100-018-0142-3 (PMC6298018; doi:10.1186/s13100-018-0142-3)
Supplement: Supplementary file 3 — Sequence verification of non-reference solo LTR or provirus alleles. (PDF 47 kb) [file 13100_2018_142_MOESM3_ESM.pdf]

Additional file 3. Sequence verification of non-reference solo LTR or provirus alleles

>S_Yadava-1_18q21.1_W2_env_partial_sequence
GGAAATCTCNCTGCACAACCCCTACTACACCCCAATTCAGCAGGAAGCAGTTAGAGCAGTTGTCAGCCAACCTCCCC
AACAGCACTTGGGTTTTCCTGTTGAGAGGGGGGACTGAGAGACAGGACTAGCTGGATTTCCTAGGCCAACTAAGAAT
CCCTAAGCCTAGCTGGGAAGGTGACCGCATCCACCTTTAAACATGGGGCTTGCAACTTAGCTCACACCGGCCAACCA
GGTAATAAAGAGAGCTCACTAAAATGCTAATCAGGCAAAAACAGCAGGTAAAAAAATAGCCAATCATCTTTCGCCTG
AGACCACAGTGGGCGGGACAATGATCAGGATATAAACCCAGGCATTCAAGCCAGCAATGGCTACCCTCTTTGGGTCC
CCTCCCTTTGTATGGGAGCTCTGTTTTCGCTCTATTAAATCTTGCAACTGCGCTCTCTTCTGGTGCATGTTTGCTAT
GGCTCGGCTCTAGCTGAGCTTTTGTTCACTGTCCACCACTGCTGTTTGCTGCCGCCGCAGACCTGCCACTGACTTCC
ATCCCTTCGGATTCGGCAGGGTGTCCTCTGTGCCCCTGATCCAGCAAGGCACCCATTGCTGCTCCTGATCAGGCTAA
AGTCTTGCCATTGTTCCTGCATGGCTAAGTGCCCAGGTTCATCCTAATTGAGCTGAACACTAGTCACTGGGTTCACA
GTTCTCTTCCGTGACCCATGGCTTCTAATAGAGCTATAACACTCACTGCATGGCCCAAGATTCCATTCCTTGGAATC
CATGAGGCCAAGAACCCCNGTCAGAGAACACGAGGCTTGCCACCATTTTGGAAGTGGCCTGCCGCCATCTTGGGAGC
TCTGGGANCAAGGACTCCCCCGGTAACACTACCATCATCTCTCATCTGATAAGTGAAATAGCCTA

>S_Yadava-2_18q21.1_W2_env_partial_sequence
AATCTCNNTGCACAACCCCTACTACACCCCAATTCAGCAGGAAGCAGTTAGAGCAGTTGTCAGCCAACCTCCCCAAC
AGCACTTGGGTTTTCCTGTTGAGAGGGGGGACTGAGAGACAGGACTAGCTGGATTTCCTAGGCCAACTAAGAATCCC
TAAGCCTAGCTGGGAAGGTGACCGCATCCACCTTTAAACATGGGGCTTGCAACTTAGCTCACACCGGCCAACCAGGT
AATAAAGAGAGCTCACTAAAATGCTAATCAGGCAAAAACAGCAGGTAAAAAAATAGCCAATCATCTTTCGCCTGAGA
CCACAGTGGGCGGGACAATGATCAGGATATAAACCCAGGCATTCAAGCCAGCAATGGCTACCCTCTTTGGGTCCCCT
CCCTTTGTATGGGAGCTCTGTTTTCGCTCTATTAAATCTTGCAACTGCGCTCTCTTCTGGTGCATGTTTGCTATGGC
TCGGCTCTAGCTGAGCTTTTGTTCACTGTCCACCACTGCTGTTTGCTGCCGCCGCAGACCTGCCACTGACTTCCATC
CCTTCGGATTCGGCAGGGTGTCCTCTGTGCCCCTGATCCAGCAAGGCACCCATTGCTGCTCCTGATCAGGCTAAAGT
CTTGCCATTGTTCCTGCATGGCTAAGTGCCCAGGTTCATCCTAATTGAGCTGAACACTAGTCACTGGGTTCACAGTT
CTCTTCCGTGACCCATGGCTTCTAATAGAGCTATAACACTCACTGCATGGCCCAAGATTCCATTCCTTGGAATCCAT
GAGGCCAAGAACCCCAGGTCAGAGAACACGAGGCTTGCCACCATTTTGGAAGTGGCCTGCCGCCATCTTGGGAGCTC
TGGGAGCAAGGACTCCCCCGGTAACACTACCATCATCTCTCATCTGAATAAGTGAAATAGCCTAC

>S_Mala-3_18q21.1_W2_env_partial_sequence
GGTAGTGTTACCGGGGGAGTCCTTGCTCCCAGAGCTCCCAAGATGGCGGCAGGCCACTTCCAAAATGGTGGCAAGCC
TCGTGTTCTCTGACCTGGGGTTCTTGGCCTCATGGATTCCAAGGAATGGAATCTTGGGCCATGCAGTGAGTGTTATA
GCTCTATTAGAAGCCATGGGTCACGGAAGAGAACTGTGAACCCAGTGACTAGTGTTCAGCTCAATTAGGATGAACCT
GGGCACTTAGCCATGCAGGAACAATGGCAAGACTTTAGCCTGATCAGGAGCAGCAATGGGTGCCTTGCTGGATCAGG
GGCACAGAGGACACCCTGCCGAATCCGAAGGGATGGAAGTCAGTGGCAGGTCTGCGGCGGCAGCAAACAGCAGTGGT
GGACAGTGAACAAAAGCTCAGCTAGAGCCGAGCCATAGCAAACATGCACCAGAAGAGAGCGCAGTTGCAAGATTTAA
TAGAGCGAAAACAGAGCTCCCATACAAAGGGAGGGGACCCAAAGAGGGTAGCCATTGCTGGCTTGAATGCCTGGGTT
TATATCCTGATCATTGTCCCGCCCACTGTGGTCTCAGGCGAAAGATGATTGGCTATTTTTTTACCTGCTGTTTTTGC
CTGATTAGCATTTTAGTGAGCTCTCTTTATTACCTGGTTGGCCGGTGTGAGCTAAGTTGCAAGCCCCATGTTTAAAG
GTGGATGCGGTCACCTTCCCAGCTAGGCTTANGGATTCTTAGTTGGCCTANGAAATCCAGCTAGTCCTGTCTCTCAG
TCCCCCCTCTCAACAGGAAAACCCAAGTGCTGTTGGGGAGGTTGGCTGACAACTGCTCTAACTGCTTCCTGCTGAAT
TGGGGTGTAGTAGGGGTTGTGCAGTTGAGATTTCCTTGGGAGGGTGCNTTGATGTCAT

>S_Kapu-1_18q21.1_W2_gag_partial_sequence
GANAGCAAGCGNAAGAGGTCCAATATTACTCACTGCTTTGGAGATCTCTTCGTGGTTACCAAAATGTCACCGGGGGT
TCCTTGCTCCCAGAGCTCCCAAGATGGCAGCAGGCCACTTCCAAGATGGTGGCAAGCCTTGGCCTCACGGATTCCAA
AGAATGGAATCTTGCACCATGCGATGAGTGTTATAGCTCTATTAGAAGCCGTGGGTCACGGAAGAGAACCATGGAAC
CCAGTGACTAGTGTTCAGCTCAATTAGGACAAATCTGGGCACTTAGCCATGCAGGAACAATGGCAAGACTTTAGCCC
GATCAGGAGCAGCAATGCGTGCCTTGCTAGATCAGGAGCACAGAGGACACCCTGCCGGATCCGAAGGGATGGAAGTC
AGCAGCGGGTCTGCGGTGGTGGCAAACAGCAGTGGTGGACAGTGAGCGAAAGCTCAGCTTGAGCCAAGCCATAACAA
ACATGCACCAGAAGAGAGCGCAGTTGCAAGATTTAATAGAGCGAAAACAGAGCTCCCATACAAAGGGAGGGGACCCA
AAGAGGGTAGCCATTGCCAGCTTGAAGGCCTGGGTTTATATCCCGATCATTGTCCCCCTCACTGTGCTCTCAGGCAA
TAGGTGATTGGCTATTTCTTTACCTCCTGTTTTTGCCTAATTAGCATTTTAGTGAGCTCTCTTTATTACCTGGTTGG
TCGGTGTGAGCTAAGTTGCAAGCCCCATGTTTAAAGGTGGATGTGGTCACCTTCCCAGCTAGGCTTAGGGATTCTTA
GTCAGCCTAGGAAATCCAGCTTGTCCTGTCTCTCAGTAGCATGAGCAATATTGGTGACAGAGAGGAAGAGCTGAATT
CAGTAGACCTGATGATGGTTGGATAG

>S_Yadava-1_18q21.1_W2_gag_partial_sequence
GCTCTTCCTCTCTGTCACCAATATTGCTCATGCTACTGAGAGACAGGACAAGCTGGATTTCCTAGGCTGACTAAGAA
TCCCTAAGCCTAGCTGGGAAGGTGACCACATCCACCTTTAAACATGGGGCTTGCAACTTAGCTCACACCGACCAACC
AGGTAATAAAGAGAGCTCACTAAAATGCTAATTAGGCAAAAACAGGAGGTAAAGAAATAGCCAATCACCTATTGCCT
GAGAGCACAGTGAGGGGGACAATGATCGGGATATAAACCCAGGCCTTCAAGCTGGCAATGGCTACCCTCTTTGGGTC
CCCTCCCTTTGTATGGGAGCTCTGTTTTCGCTCTATTAAATCTTGCAACTGCGCTCTCTTCTGGTGCATGTTTGTTA
TGGCTTGGCTCAAGCTGAGCTTTCGCTCACTGTCCACCACTGCTGTTTGCCACCACCGCAGACCCGCTGCTGACTTC
CATCCCTTCGGATCCGGCAGGGTGTCCTCTGTGCTCCTGATCTAGCAAGGCACGCATTGCTGCTCCTGATCGGGCTA
AAGTCTTGCCATTGTTCCTGCATGGCTAAGTGCCCAGATTTGTCCTAATTGAGCTGAACACTAGTCACTGGGTTCCA
TGGTTCTCTTCCGTGACCCACGGCTTCTAATAGAGCTATAACACTCATCGCATGGTGCAAGATTCCATTCTTTGGAA
TCCGTGAGGCCAAGGCTTGCCACCATCTTGGAAGTGGCCTGCTGCCATCTTGGGAGCTCTGGGAGCAAGGAACCCCC
GGTGACATTTTGGTAACCACGAAGAGATCTCCAAAGCAGTGAGTAATATTGGACCTCTTTCGCTTGCTATTCTGTCC
TATCCTTCCTTAGAATTG

>S_Mala-1_4q22.1_H8_env_partial_sequence
TGAGAAACATCGCCCCATTATCTCTCCATACCACCCCCAACACTTCAACACTATTTTATTTTTCTTATTAATATAAG
AAGACAGGAATGTCAGGCCTCTGAGCCCAAGCCTGCATATATACATCCAGATGGCCTGAAGCAAGTGAAGAATCACA
AAAKAAGTGAAAATGGCCGGTTCCTGCCTTAACTGATGACATTACCTTGTGAAATTCCTTCTCCTGGCTCAGAAGCT
CCCCCATTGAGCACCTTGTGACCCCTGCCCCTGCCGGCCAGAGAACCCCCTTTGACTGTAATTTTCCATTATCTACC
CAAATCCTGTAAAACAGCCCCACCCCTATCTCCCTTYGCTGACTCTCTTTTCTGACTCAGCCCRCCTGCACCCAGGT
GATTAAAAAGCTTTATTGCTCACACAAAGCCTGTTTGGTGGTCTCTTCACACGGATGCATGTGAAAATTATACCCCA
AGATTTTGCATTATTTTTATCTTGTCTAACTATATAATGAAAACAAATTGAAAAGACCCAATATATTAAAAGCTACT
TTATAGGAGTTTTAAAAAGAATTRTATCTATCAAGAATCTCATTATAATTGTAGATACTATGGAGGATTATGGAATA
GAGATACACCAAATATTTCCATAATAATAATGTTTTATCTGTTTTTA

>S_Madiga-2_4q22.1_H8_env_partial_sequence
CCACTCTAGGTTCCCACACCACCCCCTAATCCCGCTGGAACCAGCCCTGAGAAACATCGCCCCATTATCTCTCCATA
CCACCCCCAACACTTCAACACTATTTTATTTTTCTTATTAATATAAGAAGACAGGAATGTCAGGCCTCTGAGCCCAA
GCCTGCATATATACATCCAGATGGCCTGAAGCAAGTGAAGAATCACAAAAGAAGTGAAAATGGCCGGTTCCTGCCTT
AACTGATGACATTACCTTGTGAAATTCCTTCTCCTGGCTCAGAAGCTCCCCCATTGAGCACCTTGTGACCCCTGCCC
CTGCCCGCCAGAGAACCCCCTTTGACTGTAATTTTCCATTATCTACCCAAATCCTGTAAAACAGCCCCACCCCTATC
TCCCTTCGCTGACTCTCTTTTCTGACTCAGCCCGCCTGCACCCAGGTGATTAAAAAGCTTTATTGCTCACACAAAGC
CTGTTTGGTGGTCTCTTCACACGGATGCATGTGAAAATTATACCCCAAGATTTTGCATTATTTTTATCTTGTCTAAC
TATATAATGAAAACAAATTGAAAAGACCCAATATATTAAAAGCTACTTTATAGGAGTTTTAAAAAGAATTGTATCTA
TCAAGAATCTCATTATAATTGTAGATACTATGGAGGATTATGGAATAGAGATACACCAAATATTTCCATAATAATAA
TGTTTTATCTGTTTTTATTTTTAT

>S_Brahmin-1_5p15.31_H2_env_partial_sequence
TCTAGGTTCCCATGCCACCCCTAATCCCGCTCAAAGCAGCCTTGAGAAACATTGCTCATTATCTCTCCATACCACCC
CCAAAAAATTTTCACCATCCCAACACTTCAGCACTATTTCATTTTTCTTATTAATATAAGAAGACAGGAATATCAGG
CCTCTGAGCCCAAGCTAAGCCATCATATCCCCTGTGACCTGCACGTACACATCCAGATGGCCGGTTCCTGCCTTAAC
TGATGACATTCCACCACAAAAGAAGTGAAAATGGCCTGTTCCTGCCTTAACTGATGACATTGTCTTGTGAAATTCCT
TCTCCTGGCTCATCCTGGCTCAAAAGCTCCCCTACTGAGCACCTTGTGACCCCCACTCTGCCTGCCAGAGAACAACC
CCCTTTGACTATAATTTTCCTTTATCTACTCACATCCTATAAAATGGCCCCACCCCTATCTCCCTTCGCTGACTCTT
TTCGGACTCAGCCTGCCTGCACCCAGGTGATTAAAAGCTTTATTGCTCACACAAAACCTGTTTGATGGTCTCTTCAC
ACGGACGCGCATGAAAGTTACAACTTGAAAATATGATACACACATTAGAAAAAAAGCATGCCAATTAAATGCAATTT
ATAGAAACACAACAAAAGAAACTCAGAAAAATGATGAAAAAAGATACGGTTCACATACAGCAAGATGGTGTAGCTCC
ACTCGTGAAACACAATGGACTTTAAAACAACAAGCC

>S_Madiga-2_5p15.31_H2_env_partial_sequence
CCCACTCTAGGTTCCCATGCCACCCCTAATCCCGCTCAAAGCAGCCTTGAGAAACATTGCTCATTATCTCTCCATAC
CACCCCCAAAAAATTTTCACCATCCCAACACTTCAGCACTATTTCATTTTTCTTATTAATATAAGAAGACAGGAATA
TCAGGCCTCTGAGCCCAAGCTAAGCCATCATATCCCCTGTGACCTGCACGTACACATCCAGATGGCCGGTTCCTGCC
TTAACTGATGACATTCCACCACAAAAGAAGTGAAAATGGCCTGTTCCTGCCTTAACTGATGACATTGTCTTGTGAAA
TTCCTTCTCCTGGCTCATCCTGGCTCAAAAGCTCCCCTACTGAGCACCTTGTGACCCCCACTCTGCCTGCCAGAGAA
CAACCCCCTTTGACTATAATTTTCCTTTATCTACTCACATCCTATAAAATGGCCCCACCCCTATCTCCCTTCGCTGA

CTCTTTTCGGACTCAGCCTGCCTGCACCCAGGTGATTAAAAGCTTTATTGCTCACACAAAACCTGTTTGATGGTCTC
TTCACAYGGACGCGCATGAAAGTTACAACTTGAAAATATGATACACACATTAGAAAAAAAGCATGCCAATTAAATGC
AATTTATAGAAACACAACAAAAGAAACTCAGAAAAATGATGAAAAAAGATACGGTTCACATACAGCAAGATGGTGTA
GCTCCACTCGTGAAACACAATGGACTTTAAAACAACAAGC

>S_Madiga-2_2q34_H4_soloLTR
TAGGTGAAAGAGCTCACATAGCAGAGTATTGTGATATTAGCCATGATGTACTAACCATCTACATATGTTAGTTGATT
GTAATAATAATAAAATAATTAATTTTAAAGATATAAACTATACTTATTGTCAGGTCTCTGAGCCCAAGCCAAGCCAT
CGCATCCCCTCTGACTTGCAGGTATATGCCCAGATGGCCTGAAGTAACTGAAGAATCACAAAAGAAGTGAAAATGCC
CTGCCCCGCCTTAACTGATGACATTCCACCAAAAAAGAAGTGTAAATGGCCGGTCCTTGCCTTAAGTGATGACATTA
CCTTGTGAAAGTCTTTTTCCTGGCTCATCCTGGCTCAAAAACTCCCCCACTGAGCACCTTGCGACCCCCACTCCTGC
CCACCAGAGAACAAACCCCCTTTGACTGTAATTTTCCTTTACCTGACCAAATCTTATAAAACGGCCCCACCCCTATC
TCCCTTCTCTGACTCTCTTTTCGGACTCAGCCCGCTTGCACCCAGGTGAAATAAACAGCCATGTTGCTCACACAAAG
CCTGTTTGGTCTCTTCACACGGACGTGCATGAAACTTATTAATTACTAAAGATAAATAAGTGAAGTAACACTAGATT
CATTTTAACCTGAATTAATACAAATAAGGTGAAATGGTTGGCCTACTGATTGTCTATAAGGAGCAAACCTATAAAAT
ATCAAGTATTGTCACTGCTTGAATATGCAAATGAAAGCTGTGAATAATAATCACGTGGTCAGGGAGAGAAATAAGTT
TAAAGATATTAATAAATCTAGAGCACTTTCACATTATGTCAATGTAGTTAAAAGGATGAAAAGAAAATGTTTATCTT
CCAGGA

>S_Luhya-2_3p14.3_H1_soloLTR
TTCTCTTTGGGAATGTCAGGCCTCTGAGCCCAAGCCAAGCCATCGCATCCCCTATGACATGCACGTACACGCCCAGA
TGGCCTGAAGTAACTGAAGAATCACAAAAGAAGTGAATATGCCCTGCCCCACCTTAACTGATGACATTCCACCACAA
AAGAAGTGTAAATGGCCAGTCCTTGCCTTAACTGATGACATTACCTTGTGAAAGTCCTTTTCCTGGCTCATCCTGGC
TCAAAAAGCACCCCCACTGAGCACCTTGCGACCCCCCGCTCCTACCCGCCAGAGAACAAACCCCCTTTGACTGTAAT
TTTCCTTTACCTACCCAAATCCTATAAAACGGCCCCACCCTTATCTCCCTTCGCTGACTCTCTTTTCCGACTCAGCC
CGCCTGCACCCAGGTGAAATAAACAGCCTTGTTGCTCACACAAAGCCTGTTTGGTGGTCTCTTCACACAGACGCGCA
TGAAAGGGAAGACATACAAAAACAAGGTAAATAAGTAAACTACGTTATATGTTTGATAATGGTGATGTTAAGGGTGG
GGAAAGAAGAAAGCAAAGAAGGATAAGAAATGGGAGGGGGCAATTCTAGAAAC

>S_Luhya-2_5p13.3_K2_soloLTR
TCCATCATATAAACAGAACTGTGGGGAAAAGCAAGAGAGATCAGATTGTTACTGTGTCTGTGTAGAAAGAAGTAGAC
ATAGGAGACTCCATTTTGTTATGTACTAAGAAAAATTCTTCTGCCTTGAGATTCTGTGACCTTACCCCCAACCCCGT
GCTCTCTGAAACATGTGCTGTGTCAACTCAGAGTTGAATGGATTAAGGGCRGTGCAAGATGTGCTTTGTTAAACAGA
TGCTTGAAGGCAGCATGCTCCTTAAGAGTCATCACCACTCCCTCATCTCAAGTACCCAGGGACACAAAAACTGCGGA
AGGCYGCAGGGACCTCTGCCTAGGAAAGCCAGGTATTGTCCAAGGTTTCATAGTCTGAAATATGGCCTCGTGGGAAG
GGAAAGACCTGACTGTCCCCCAGCCCGACACCCGTAAAGGGTCTGTGCTGAGGAGGATTAGTATAAGAGGAAGGCAT
GCCTCTTGCAGTTGAGACAAGAGGAAGGCATCTGTCTCCTGCCTGTCCCTGGGCAATRGAATGTCTCGKTATAAAAC
CCGATTGTATGCTCCATCTACTGAGATAGGGAAAAACYGCCTTAGGGCTGGAGGTGGGACCTGCGGGCAGCAATACT
GCTTTGTAAAGCATTGAGATGTTTATGTGTATGCATATCTAAAAGCACAGCACTTAATCCTTTACATTGTCTATGAT
GCAAAGACCTTTGTTCACGTGTTTGTCTGCTGACCCTCTCCCACATTGTCTTGTGACCCTGACACATCCCCCTCTTT
GAGAAACACCCACGAATGATCAATAAATACTAAKGGAACTCAGAGGCTGGCGGGATCCTCCATATGCTGAACGCTGG
TTCCCCGGATCCCCTTATTTCTTTCTCTATACTTTGTCTCTGTGTCTTTTTCTTTCCTAAGTCTCTCATTCCACCTT
ACGAGAAACACCCACAGGTGTGGAGGGGCAACCCACCCCTACACAGAACCAATGACAAAAACCACATGATTATCTCA
ATAGATGCAGAAAAGGCCTTCGACAAAATTCAATAGCCATTAATGCTAAAAACTCTCAATAAACTAGGTATCGATGG
AACATATCTCAAAATAATAAGCGTTATTTATGACAAACYCACAGCTAATATCATACTGAATGTGCAAAAACTGGAAG
CATTCCCTTTGAAAACTGGCAAAAGACAAGTATGCCCTCTCTACCACTATTCAACATAATGTTGTTAGTTCTGGCAA
GGGCAATGAGGCCAGAGAAAGAAATAAAGGGTATTTAATTAGGAAAAGAGGAAGTCAAATTGTCCCTGTTTGCAGAT
GACATGATTGTATATTTAGAAAACCCCATTGTCTCAGCCCAAAATCTCCTTAAGCTGATAAGCAACTTCAGCAAAAT
CTCAGGATACAAAATCAATGTGCAAAAATCACAACCATTCCTATACATCAATAACAGACAAACAGAGAGCCAAATCA
TGAGTGAACTCCCATTCACAATTGCTACAAAGTGAATAAAGTACCTAGGAATCCAACTTCCAAGGGATGTGAAGGAC
CTCTTCAAGGAGAACTACAAACCACTGCTCAACAAAATAAAAGAGGACACAAACAAATGGAAGAATATTCCATGCTC
ATGGATAGGAAGAATCAATATTGTGAAAATGGCTATACAGGTCAAGGTAATTTATAGATTCAATGCCATCCCCATCA
AGCTACCAATGACTTTCTTCACAGAATTGGGAAAACTACTTTAAAGTTCATATGGAACCAAAAAA
