# Supplementary material for: Variation in proviral content among human genomes mediated by LTR recombination
Source: Mob DNA. 2018 Dec 18;9:36. doi: 10.1186/s13100-018-0142-3 (PMC6298018; doi:10.1186/s13100-018-0142-3)
Supplement: Supplementary file 4 — Comparison of predictions of solo LTR and provirus from findsoloLTR and findprovirus pipelines to PCR based genotypes for solo LTR (S) and provirus (P) polymorphisms. (PDF 168 kb) [file 13100_2018_142_MOESM4_ESM.pdf]

Additional file 4: Comparison of predictions of solo LTR and provirus from *findsoloLTR* and *findprovirus* pipelines to PCR based genotypes for solo LTR (S) and provirus (P) polymorphisms

18q21.1_W2 (Ref. allele: Solo LTR)	Individuals	S_Mala-2	S_Mala-3	S_Kapu-1	S_Kapu-2	S_Yadava-1	S_Yadava-2	S_Relli-1
	findprovirus		P	P	P	P	P	P
	PCR	S/S	S/P	S/P	S/P	S/P	P/P	S/P

4q22.1_H8 (Ref. allele: Solo LTR)	Individuals	S_Madiga-2	S_Kapu-1	S_Kapu-2	S_Brahmin-2	S_Mala-2	S_Yadava-1
	findprovirus	P	P	P	P	P	P
	PCR	P/P	S/P	S/P	S/P	P/P	S/P

5p15.31_H2 (Ref. allele: Solo LTR)	Individuals	S_Madiga-2	S_Yadava-1	S_Brahmin-1	S_Brahmin-2	S_Mala-2	S_Kapu-1
	findprovirus	P*	P*			P*	
	PCR	P	P	P	P	P	P

5p13.3_K2 (Ref. allele: Provirus)	Individuals	S_Brahmin-1	S_Brahmin-2	S_Relli-1	S_Irula-2	S_Yadava-1	S_Yadava-2	S_Luhya-2
	findsoloLTR	$S^{\$}$	$S^{\$}$	$S^{\$}$	$S^{\$}$		$S^{\$}$	$S^{\$}$
	PCR	S/P	S/P	S/S	S/P	S/P	S/P	S/P

2q34_H4 (Ref. allele: Provirus)	Individuals	S_Madiga-2	S_Kapu-1	S_Kapu-2	S_Mala-2	S_Mala-3
	findsoloLTR	S				
	PCR	S/P	P/P	P/P	P/P	P/P

3p14.3_H1 (Ref. allele: Provirus)	Individuals	S_Luhya-2	S_Madiga-2	S_Mala-2	S_Mala-3	S_Yadava-1	S_Brahmin-1	S_Kapu-1
	findsoloLTR	S						
	PCR	S/P	P/P	P/P	P/P	P/P	P/P	P/P

Footnotes: 'S' denotes solo LTR and 'P' denotes Provirus. *no of discordant reads identified were less than 4. $ The Percentage Read Depth criteria in those individuals are between 51-60.
