# Supplementary material for: Variation in proviral content among human genomes mediated by LTR recombination
Source: Mob DNA. 2018 Dec 18;9:36. doi: 10.1186/s13100-018-0142-3 (PMC6298018; doi:10.1186/s13100-018-0142-3)
Supplement: Supplementary file 5 — Alignment of 18q21.1_W2 solo LTR and provirus with HERV-W consensus. (PDF 707 kb) [file 13100_2018_142_MOESM5_ESM.pdf]

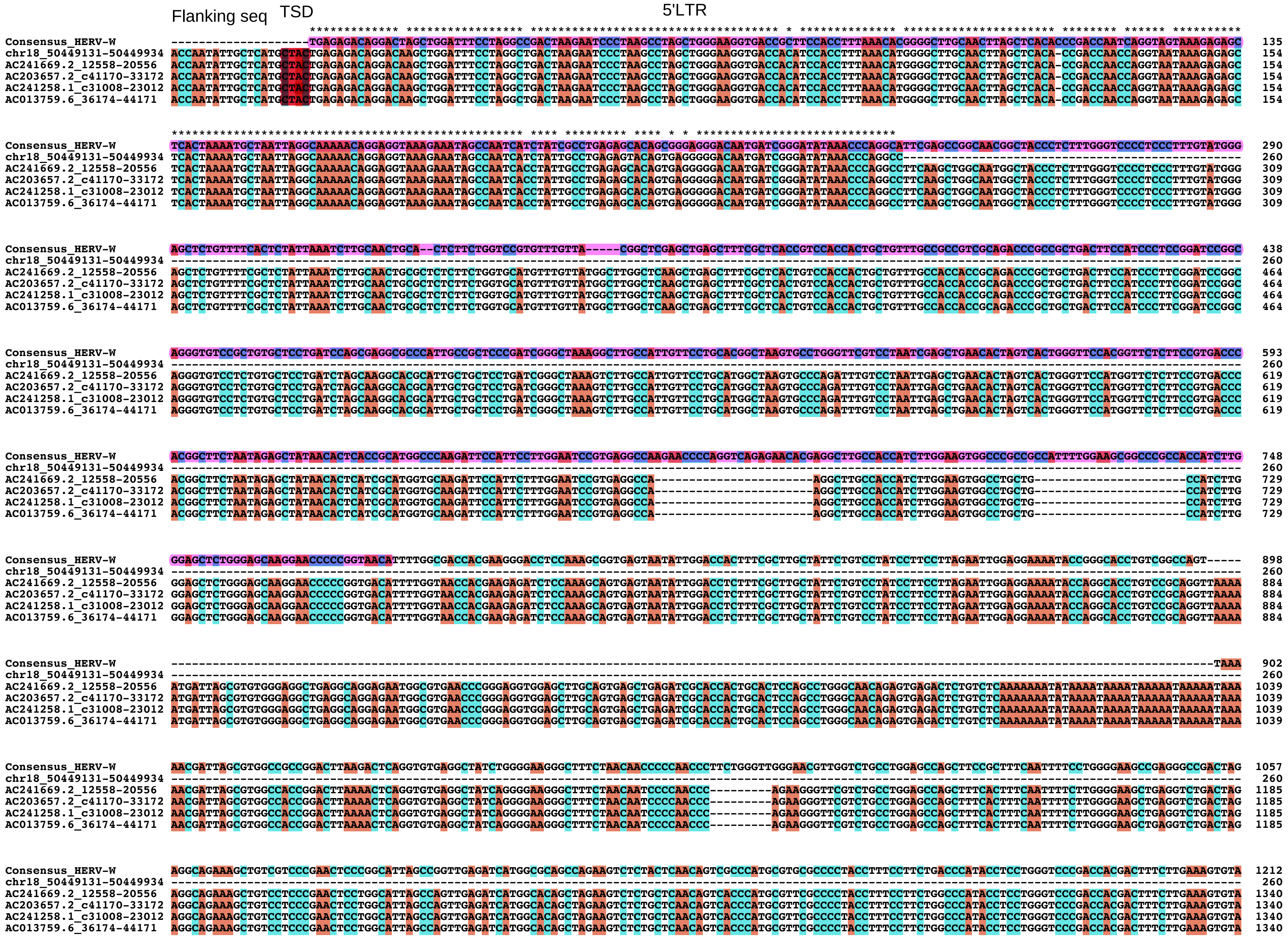

Flanking seq
TSD
5'LTR

```
Consensus_HERV-W             GCCCCAAAATTCTCCTTACCTCTGAATCTACTTCCTCTGATCCCTGCCTCCTAGGTACTAATGGTTCAGACTTTCATTTCCTCTAGCAAGTTGTATCTCCAAAGGGATCTAAGGAAGCTCTACGCTGCGTCCTTAGGCACCTAGGCTATGAACCC  1367
chr18_50449131-50449934      -----------------------------------------------------------------------------------------------------------------------------------------------------------  260
AC241669.2_12558-20556       GCCCCAAAATTCTCCTTACTTCTGAATCTACTTCCCCTGATCCCTACCTCCTAGGTACTAATGGTTCAGACTTTCATTTCATCTAGCAAGTTGTATCTCCAAAGGGATCTAAGGAAGCTCTATGCTGCATCCTTAGGCATCTAGGCTATAAATCC  1495
AC203657.2_c41170-33172      GCCCCAAAATTCTCCTTACTTCTGAATCTACTTCCCCTGATCCCTACCTCCTAGGTACTAATGGTTCAGACTTTCATTTCATCTAGCAAGTTGTATCTCCAAAGGGATCTAAGGAAGCTCTATGCTGCATCCTTAGGCATCTAGGCTATAAATCC  1495
AC241258.1_c31008-23012      GCCCCAAAATTCTCCTTACTTCTGAATCTACTTCCCCTGATCCCTACCTCCTAGGTACTAATGGTTCAGACTTTCATTTCATCTAGCAAGTTGTATCTCCAAAGGGATCTAAGGAAGCTCTATGCTGCATCCTTAGGCATCTAGGCTATAAATCC  1495
AC013759.6_36174-44171       GCCCCAAAATTCTCCTTACTTCTGAATCTACTTCCCCTGATCCCTACCTCCTAGGTACTAATGGTTCAGACTTTCATTTCATCTAGCAAGTTGTATCTCCAAAGGGATCTAAGGAAGCTCTATGCTGCATCCTTAGGCATCTAGGCTATAAATCC  1495

Consensus_HERV-W             AGGGAGTCTTGTCCCTGGTGTCCCTCCCAATTTAGGCATACAGCTCTCGACATGGGCAGTTATGTGGGACCCGTTCCCCACCACCCTTGCCAGGGCCCCAAGTTTGTAAATGGCTAAGGGAAAAGA----GAGACAGAGGAGAGAGAGAGAGACG  1518
chr18_50449131-50449934      -----------------------------------------------------------------------------------------------------------------------------------------------------------  260
AC241669.2_12558-20556       AGGGACTCTTATTCCTGATGTCCCTCCTGATTTAGGTATACAGCTCTCGACATGGGCAGTTATGTGGGACCCATTCCCTACCACCCTTGCCAGGGCCCCAAGCTTGT-AATGGCGGAGGAGAGAGAGCGCGAGACAGAGGAGAGAGCGTGAGATG  1649
AC203657.2_c41170-33172      AGGGACTCTTATTCCTGATGTCCCTCCTGATTTAGGTATACAGCTCTCGACATGGGCAGTTATGTGGGACCCATTCCCTACCACCCTTGCCAGGGCCCCAAGCTTGT-AATGGCGGAGGAGAGAGAGCGCGAGACAGAGGAGAGAGCGTGAGATG  1649
AC241258.1_c31008-23012      AGGGACTCTTATTCCTGATGTCCCTCCTGATTTAGGTATACAGCTCTCGACATGGGCAGTTATGTGGGACCCATTCCCTACCACCCTTGCCAGGGCCCCAAGCTTGT-AATGGCGGAGGAGAGAGAGCGCGAGACAGAGGAGAGAGCGTGAGATG  1649
AC013759.6_36174-44171       AGGGACTCTTATTCCTGATGTCCCTCCTGATTTAGGTATACAGCTCTCGACATGGGCAGTTATGTGGGACCCATTCCCTACCACCCTTGCCAGGGCCCCAAGCTTGT-AATGGCGGAGGAGAGAGAGCGCGAGACAGAGGAGAGAGCGTGAGATG  1649

Consensus_HERV-W             GAGGAGAGAGAGAGAGAGACAGAGAGGAGAGAGAGACAGGAGAGAGACAGAAGAGAGAGAGAGACAGAGAGGAGAG------AGAGAGAGTCAAAGAGAGAAAGAAAGAGAWAGA----------------------------------------  1627
chr18_50449131-50449934      -----------------------------------------------------------------------------------------------------------------------------------------------------------  260
AC241669.2_12558-20556       GAG--GAGATAGAGCGAGATGGAGGAGAGAGAGTGACACGG-------AGGACAGAGAGTGAGATGGAGGAGAGAGAGACAAAGAGGGAGTCAAAGAGAAAAAGAAAGATATAAATAGTAAAAAAAAAAAAACAAAAAAACAAAAAAACAAAAAA  1795
AC203657.2_c41170-33172      GAG--GAGATAGAGCGAGATGGAGGAGAGAGAGTGACACGG-------AGGACAGAGAGTGAGATGGAGGAGAGAGAGACAAAGAGGGAGTCAAAGAGAAAAAGAAAGATATAAATAGTAAAAAAAAAAAAACAAAAAAACAAAAAAACAAAAAA  1795
AC241258.1_c31008-23012      GAG--GAGATAGAGCGAGATGGAGGAGAGAGAGTGACACGG-------AGGACAGAGAGTGAGATGGAGGAGAGAGAGACAAAGAGGGAGTCAAAGAGAAAAAGAAAGATATAAATAGT--AAAAAAAAAAACAAAAAAACAAAAAAACAAAAAA  1793
AC013759.6_36174-44171       GAG--GAGATAGAGCGAGATGGAGGAGAGAGAGTGACACGG-------AGGACAGAGAGTGAGATGGAGGAGAGAGAGACAAAGAGGGAGTCAAAGAGAAAAAGAAAGATATAAATAGT-AAAAAAAAAAAACAAAAAAACAAAAAAACAAAAAA  1794

Consensus_HERV-W             --AATAGTAAAGAAAAAACAGTGTGCCCTATTCCTTTAAAAGCCAGGGTAAATTTAAAACCTATAATTGATAATTGAAGGTCTTCTCCGTGACCCTATAACACTCCAATACCACCTTGTTGTCAGTGTAAACAAGGGCGTAGCCCGAAAGCACTG  1780
chr18_50449131-50449934      -----------------------------------------------------------------------------------------------------------------------------------------------------------  260
AC241669.2_12558-20556       CCAAAAAAACAAAAAAAAAAGTGTGCCCTATTCCTTTAAAAGCCAGGGTAAATTTCAAACCTATAATTGATAATTGAAGGTCTTCTCCATGACGCTATAATACTCCAATACTACT---TTGTCAGTGTAAACAAGGGAGTAGCCTGAAAACACGG  1947
AC203657.2_c41170-33172      CCAAAAAAACAAAAAAAAAAGTGTGCCCTATTCCTTTAAAAGCCAGGGTAAATTTCAAACCTATAATTGATAATTGAAGGTCTTCTCCATGACGCTATAATACTCCAATACTACT---TTGTCAGTGTAAACAAGGGAGTAGCCTGAAAACACGG  1947
AC241258.1_c31008-23012      CCAAAAAAACAAAAAAAAAAGTGTGCCCTATTCCTTTAAAAGCCAGGGTAAATTTCAAACCTATAATTGATAATTGAAGGTCTTCTCCATGACGCTATAATACTCCAATACTACT---TTGTCAGTGTAAACAAGGGAGTAGCCTGAAAACACGG  1945
AC013759.6_36174-44171       CCAAAAAAACAAAAAAAAAAGTGTGCCCTATTCCTTTAAAAGCCAGGGTAAATTTCAAACCTATAATTGATAATTGAAGGTCTTCTCCATGACGCTATAATACTCCAATACTACT---TTGTCAGTGTAAACAAGGGAGTAGCCTGAAAACACGG  1946

Consensus_HERV-W             AGGCCACTGACAACCCGTAGCCTTCCTATCAAAAATCCTTAACCCAGTAACCCGCGGATGGCCCAAATGCATTCAGTCNGTAGCGGCAACTGCTTTGCTAACAGAAGAAAGTAGAAAAGTAACTTTTAGAGGAAACCTCATTGTGAGCACACCTC  1935
chr18_50449131-50449934      -----------------------------------------------------------------------------------------------------------------------------------------------------------  260
AC241669.2_12558-20556       AGACCGCTGACAACCCATAGCTTTCCTATCAAAAATCCTTAACCCAGTAACCCGCGG---------AGGCATTCAATCTGTAGCAGCAACTGCTTTGCTAACAAAAGAAAGTAGAAAAGTAACTTTTAGAGGAAATGTCATTGTGAGCACACCTC  2093
AC203657.2_c41170-33172      AGACCGCTGACAACCCATAGCTTTCCTATCAAAAATCCTTAACCCAGTAACCCGCGG---------AGGCATTCAATCTGTAGCAGCAACTGCTTTGCTAACAAAAGAAAGTAGAAAAGTAACTTTTAGAGGAAATGTCATTGTGAGCACACCTC  2093
AC241258.1_c31008-23012      AGACCGCTGACAACCCATAGCTTTCCTATCAAAAATCCTTAACCCAGTAACCCGCGG---------AGGCATTCAATCTGTAGCAGCAACTGCTTTGCTAACAAAAGAAAGTAGAAAAGTAACTTTTAGAGGAAATGTCATTGTGAGCACACCTC  2091
AC013759.6_36174-44171       AGACCGCTGACAACCCATAGCTTTCCTATCAAAAATCCTTAACCCAGTAACCCGCGG---------AGGCATTCAATCTGTAGCAGCAACTGCTTTGCTAACAAAAGAAAGTAGAAAAGTAACTTTTAGAGGAAATGTCATTGTGAGCACACCTC  2092

Consensus_HERV-W             ACCAGTTCAGAATTATTCTAAGTCAAAAAAGCAAAAAGGTAGCTTACTAACTCAAAAATCTTAAAGTATGGGGCTATTCTGTTAGAAAAAGGTAATTTAACACCAACCACTGANAATTCCCTTAACCCAGCAGATTTCCTAACAGGGGATTTAAA  2090
chr18_50449131-50449934      -----------------------------------------------------------------------------------------------------------------------------------------------------------  260
AC241669.2_12558-20556       ACCAGTTCAGAATTATTCTAAGTCAAAAAAGCAAAAAAGTAGCTTACTAACTCAAAAATCTTAAAGTATGGGGCTATTCTGTTAGAAAAAGTTAATTTAACACCAACCACTGATAATTCCCCTAACCCAGCCAATTTCCTAACAGGGGATTTAAA  2248
AC203657.2_c41170-33172      ACCAGTTCAGAATTATTCTAAGTCAAAAAAGCAAAAAAGTAGCTTACTAACTCAAAAATCTTAAAGTATGGGGCTATTCTGTTAGAAAAAGTTAATTTAACACCAACCACTGATAATTCCCCTAACCCAGCCAATTTCCTAACAGGGGATTTAAA  2248
AC241258.1_c31008-23012      ACCAGTTCAGAATTATTCTAAGTCAAAAAAGCAAAAAAGTAGCTTACTAACTCAAAAATCTTAAAGTATGGGGCTATTCTGTTAGAAAAAGTTAATTTAACACCAACCACTGATAATTCCCCTAACCCAGCCAATTTCCTAACAGGGGATTTAAA  2246
AC013759.6_36174-44171       ACCAGTTCAGAATTATTCTAAGTCAAAAAAGCAAAAAAGTAGCTTACTAACTCAAAAATCTTAAAGTATGGGGCTATTCTGTTAGAAAAAGTTAATTTAACACCAACCACTGATAATTCCCCTAACCCAGCCAATTTCCTAACAGGGGATTTAAA  2247

Consensus_HERV-W             TCTTAATTACCATACAAAGGTCCGACCAGACCTAGGAGGAACTCCCTTCAGGACAGGACGATAGATGGTTCCTCCCAGGTGATTGAGGAAAAAAACCACAATGGGTATTCAGTAATTGATAGGGAGACTCTTGTGGAAGCAGAGTTAGAAAAATT  2245
chr18_50449131-50449934      -----------------------------------------------------------------------------------------------------------------------------------------------------------  260
AC241669.2_12558-20556       TCTTAATTACCATACAAAGGCCTGACCAGACCTAGGAGGAACTCCCTTCAGGACAGGACTATAGATGGTCCCTCCCAGGTGACTGAGG-AAAAAACCACAATGGGTATTCAGTAATTGATAGGGAGACTCTTGTGGAAGCAGAGTTAGAAAAATT  2402
AC203657.2_c41170-33172      TCTTAATTACCATACAAAGGCCTGACCAGACCTAGGAGGAACTCCCTTCAGGACAGGACTATAGATGGTCCCTCCCAGGTGACTGAGG-AAAAAACCACAATGGGTATTCAGTAATTGATAGGGAGACTCTTGTGGAAGCAGAGTTAGAAAAATT  2402
AC241258.1_c31008-23012      TCTTAATTACCATACAAAGGCCTGACCAGACCTAGGAGGAACTCCCTTCAGGACAGGACTATAGATGGTCCCTCCCAGGTGACTGAGG-AAAAAACCACAATGGGTATTCAGTAATTGATAGGGAGACTCTTGTGGAAGCAGAGTTAGAAAAATT  2400
AC013759.6_36174-44171       TCTTAATTACCATACAAAGGCCTGACCAGACCTAGGAGGAACTCCCTTCAGGACAGGACTATAGATGGTCCCTCCCAGGTGACTGAGG-AAAAAACCACAATGGGTATTCAGTAATTGATAGGGAGACTCTTGTGGAAGCAGAGTTAGAAAAATT  2401

Consensus_HERV-W             GCCTAATAATTGGTCTCCTCAAACGTGCGAGCTGTTTGCACTCAGCCAAGCCTTAAAGTACTTACAGAATCAAAAGACTATCTCAATCCTGACTCAAAAGGTTACCTACACCCTCTCTGAAACGAATTTGCATAAGAACTGTTGTTTATGGGAAT  2400
chr18_50449131-50449934      -----------------------------------------------------------------------------------------------------------------------------------------------------------  260
AC241669.2_12558-20556       GCCTAATAATTGGTCTCCTCAAACGTGCAAGCTGTTTGCACTCAGCCAAGCCTTAAAGTACTTACAGAATCAAAAGACTATCTCAGTCCTGACTCAAAACCTTACTTACACCTTCTCTGAAACGAATTTGCATAAAAACTGTTGTTTATGGGAAT  2557
AC203657.2_c41170-33172      GCCTAATAATTGGTCTCCTCAAACGTGCAAGCTGTTTGCACTCAGCCAAGCCTTAAAGTACTTACAGAATCAAAAGACTATCTCAGTCCTGACTCAAAACCTTACTTACACCTTCTCTGAAACGAATTTGCATAAAAACTGTTGTTTATGGGAAT  2557
AC241258.1_c31008-23012      GCCTAATAATTGGTCTCCTCAAACGTGCAAGCTGTTTGCACTCAGCCAAGCCTTAAAGTACTTACAGAATCAAAAGACTATCTCAGTCCTGACTCAAAACCTTACTTACACCTTCTCTGAAACGAATTTGCATAAAAACTGTTGTTTATGGGAAT  2555
AC013759.6_36174-44171       GCCTAATAATTGGTCTCCTCAAACGTGCAAGCTGTTTGCACTCAGCCAAGCCTTAAAGTACTTACAGAATCAAAAGACTATCTCAGTCCTGACTCAAAACCTTACTTACACCTTCTCTGAAACGAATTTGCATAAAAACTGTTGTTTATGGGAAT  2556

Consensus_HERV-W             GCATCTTGATGGGGCAGCTGGGTTGTTATGAAATACTCAGGAACCCAGCCCAGCTCTAGGACTCACCCCTGAGCGCAAAGGCAATGTTGGGCACGCTGGTAAAGGACCACTAGAATCCAGCAGCCCGGACCCCTTTCTTTGTGGTCAAGAAAGGC  2555
chr18_50449131-50449934      -----------------------------------------------------------------------------------------------------------------------------------------------------------  260
AC241669.2_12558-20556       GCATCTTGATGGGGCAGGTGGGTTGTTATGAAATACTCAGGAACCTAGCCTGGCTCTAGGACTCACCCCTGAGTGCAAAGGCGATGTTGGACATGCTGGTAAAGGAACACTAGAATCCAGTAGCCCAGACCCCTTTCTTTGTGGTCAAGAGAGGC  2712
AC203657.2_c41170-33172      GCATCTTGATGGGGCAGGTGGGTTGTTATGAAATACTCAGGAACCTAGCCTGGCTCTAGGACTCACCCCTGAGTGCAAAGGCGATGTTGGACATGCTGGTAAAGGAACACTAGAATCCAGTAGCCCAGACCCCTTTCTTTGTGGTCAAGAGAGGC  2712
AC241258.1_c31008-23012      GCATCTTGATGGGGCAGGTGGGTTGTTATGAAATACTCAGGAACCTAGCCTGGCTCTAGGACTCACCCCTGAGTGCAAAGGCGATGTTGGACATGCTGGTAAAGGAACACTAGAATCCAGTAGCCCAGACCCCTTTCTTTGTGGTCAAGAGAGGC  2710
AC013759.6_36174-44171       GCATCTTGATGGGGCAGGTGGGTTGTTATGAAATACTCAGGAACCTAGCCTGGCTCTAGGACTCACCCCTGAGTGCAAAGGCGATGTTGGACATGCTGGTAAAGGAACACTAGAATCCAGTAGCCCAGACCCCTTTCTTTGTGGTCAAGAGAGGC  2711
```

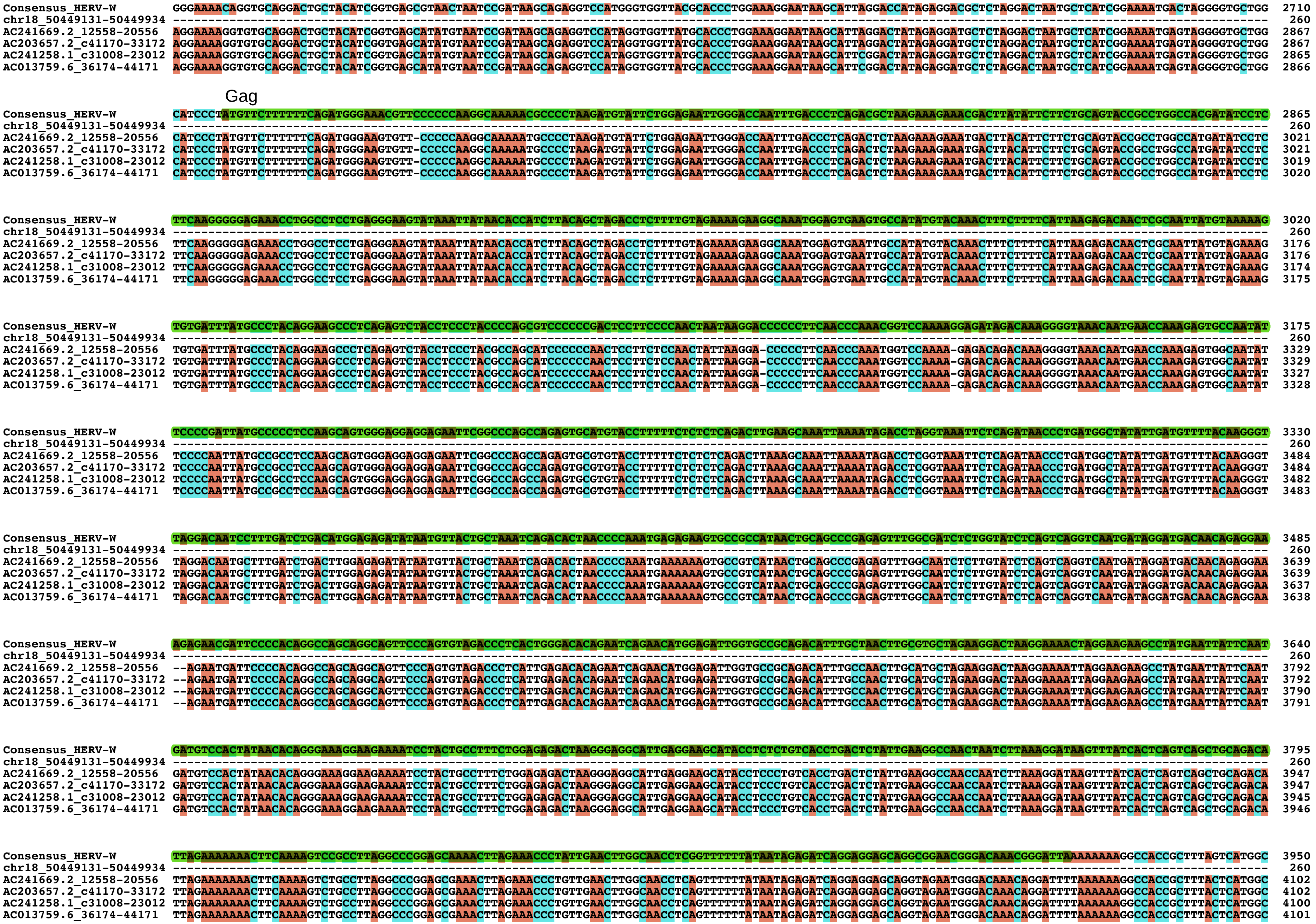
Gag
Consensus_HERV-W 2710
chr18_50449131-50449934 260
AC241669.2_12558-20556 2867
AC203657.2_c41170-33172 2867
AC241258.1_c31008-23012 2865
AC013759.6_36174-44171 2866
Consensus_HERV-W 2865
chr18_50449131-50449934 260
AC241669.2_12558-20556 3021
AC203657.2_c41170-33172 3021
AC241258.1_c31008-23012 3019
AC013759.6_36174-44171 3020
Consensus_HERV-W 3020
chr18_50449131-50449934 260
AC241669.2_12558-20556 3176
AC203657.2_c41170-33172 3176
AC241258.1_c31008-23012 3174
AC013759.6_36174-44171 3175
Consensus_HERV-W 3175
chr18_50449131-50449934 260
AC241669.2_12558-20556 3329
AC203657.2_c41170-33172 3329
AC241258.1_c31008-23012 3327
AC013759.6_36174-44171 3328
Consensus_HERV-W 3330
chr18_50449131-50449934 260
AC241669.2_12558-20556 3484
AC203657.2_c41170-33172 3484
AC241258.1_c31008-23012 3482
AC013759.6_36174-44171 3483
Consensus_HERV-W 3485
chr18_50449131-50449934 260
AC241669.2_12558-20556 3639
AC203657.2_c41170-33172 3639
AC241258.1_c31008-23012 3637
AC013759.6_36174-44171 3638
Consensus_HERV-W 3640
chr18_50449131-50449934 260
AC241669.2_12558-20556 3792
AC203657.2_c41170-33172 3792
AC241258.1_c31008-23012 3790
AC013759.6_36174-44171 3791
Consensus_HERV-W 3795
chr18_50449131-50449934 260
AC241669.2_12558-20556 3947
AC203657.2_c41170-33172 3947
AC241258.1_c31008-23012 3945
AC013759.6_36174-44171 3946
Consensus_HERV-W 3950
chr18_50449131-50449934 260
AC241669.2_12558-20556 4102
AC203657.2_c41170-33172 4102
AC241258.1_c31008-23012 4100
AC013759.6_36174-44171 4101

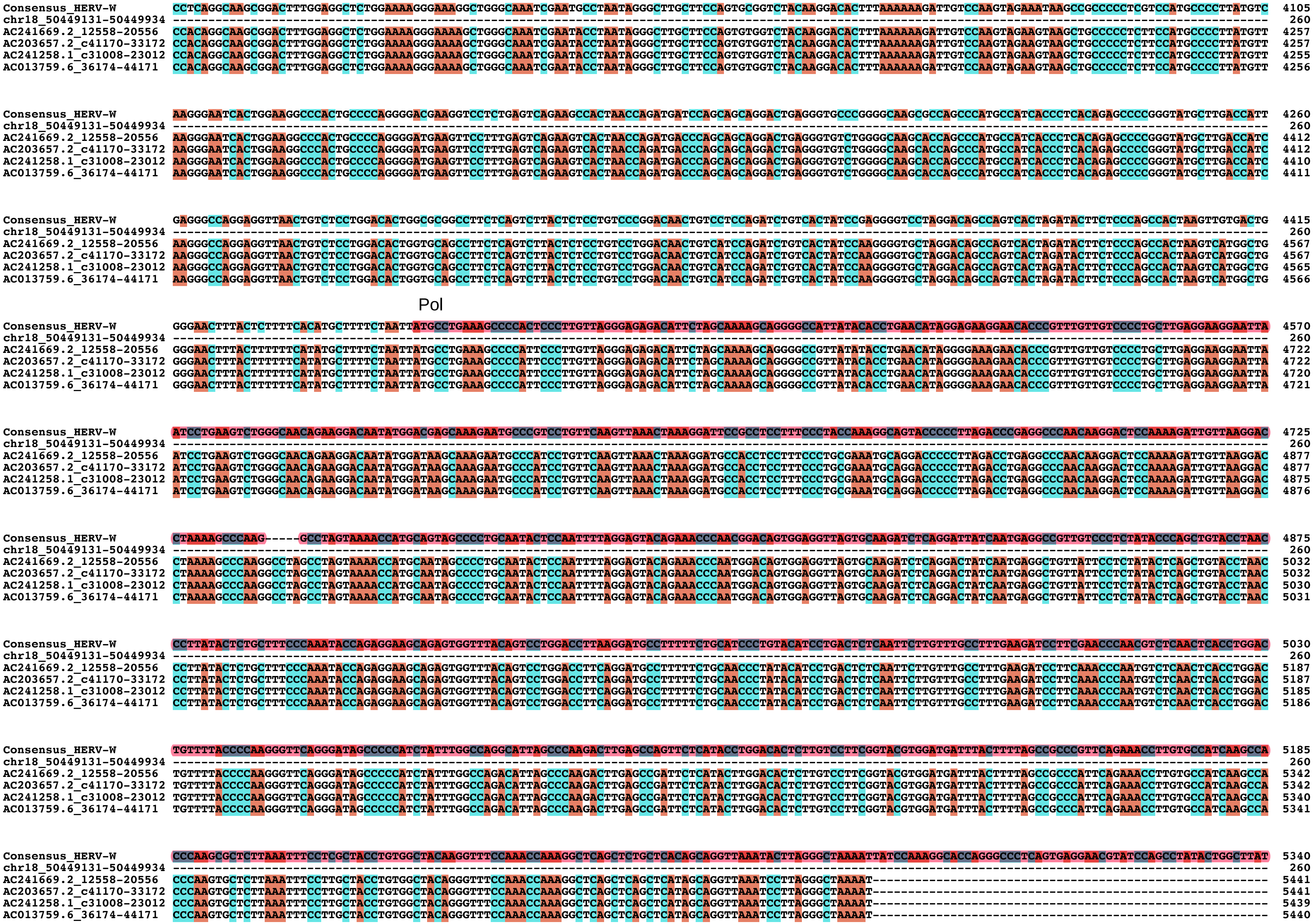
Pol
Consensus_HERV-W
chr18_50449131-50449934
AC241669.2_12558-20556
AC203657.2_c41170-33172
AC241258.1_c31008-23012
AC013759.6_36174-44171

```
Consensus_HERV-W           CCTCATCCCAAAACCCTAAAGCAACTAAGAGGGTTCCTTGGCATAACAGGTTTCTGCCGAATATGGATTCCCAGGTACGGCGAAATAGCCAGGCCATTATATACACTAATTAAGGAAACTCAGAAAGCCAATACCCATTTAGTAAGATGGACACC 5495
chr18_50449131-50449934    ----------------------------------------------------------------------------------------------------------------------------------------------------------- 260
AC241669.2_12558-20556     ----------------------------------------------------------------------------------------------------------------------------------------------------------- 5441
AC203657.2_c41170-33172    ----------------------------------------------------------------------------------------------------------------------------------------------------------- 5441
AC241258.1_c31008-23012    ----------------------------------------------------------------------------------------------------------------------------------------------------------- 5439
AC013759.6_36174-44171     ----------------------------------------------------------------------------------------------------------------------------------------------------------- 5440

Consensus_HERV-W           TGAAGCAGAAGCGGCTTTCCAGGCCCTAAAGAAGGCCCTAACCCAAGCCCCAGTGTTAAGCTTGCCAACGGGGCAAGACTTTTCTTTATATGTCACAGAAAAAACAGGAATAGCTCTAGGAGTCCTTACACAGGTCCGAGGGACGAGCTTGCAAC 5650
chr18_50449131-50449934    ----------------------------------------------------------------------------------------------------------------------------------------------------------- 260
AC241669.2_12558-20556     ----------------------------------------------------------------------------------------------------------------------------------------------------------- 5441
AC203657.2_c41170-33172    ----------------------------------------------------------------------------------------------------------------------------------------------------------- 5441
AC241258.1_c31008-23012    ----------------------------------------------------------------------------------------------------------------------------------------------------------- 5439
AC013759.6_36174-44171     ----------------------------------------------------------------------------------------------------------------------------------------------------------- 5440

Consensus_HERV-W           CCGTGGCATACCTGAGTAAGGAAATTGATGTAGTGGCAAAGGGTTGGCCTCATTGTTTACGGGTAGTGGCGGCAGTAGCAGTCTTAGTATCTGAAGCAGTTAAAATAATACAGGGAAGAGATCTTACTGTGTGGACATCTCATGATGTGAACGGC 5805
chr18_50449131-50449934    ----------------------------------------------------------------------------------------------------------------------------------------------------------- 260
AC241669.2_12558-20556     ----------------------------------------------------------------------------------------------------------------------------------------------------------- 5441
AC203657.2_c41170-33172    ----------------------------------------------------------------------------------------------------------------------------------------------------------- 5441
AC241258.1_c31008-23012    ----------------------------------------------------------------------------------------------------------------------------------------------------------- 5439
AC013759.6_36174-44171     ----------------------------------------------------------------------------------------------------------------------------------------------------------- 5440

Consensus_HERV-W           ATACTCACTGCTAAAGGAGACTTGTGGCTGTCAGACAACCGTTTACTTAAATATCAGGCTCTATTACTTGAAGGGCCAGTGCTGCGACTGCGCACTTGTGCAACTCTTAACCCAGCCACATTTCTTCCAGACAATGAAGAAAAGATAGAACATAA 5960
chr18_50449131-50449934    ----------------------------------------------------------------------------------------------------------------------------------------------------------- 260
AC241669.2_12558-20556     ----------------------------------------------------------------------------------------------------------------------------------------------------------- 5441
AC203657.2_c41170-33172    ----------------------------------------------------------------------------------------------------------------------------------------------------------- 5441
AC241258.1_c31008-23012    ----------------------------------------------------------------------------------------------------------------------------------------------------------- 5439
AC013759.6_36174-44171     ----------------------------------------------------------------------------------------------------------------------------------------------------------- 5440

Consensus_HERV-W           CTGTCAACAAGTAATTGCTCAAACCTACGCCGCTCGAGGGGACCTTTTAGAGGTTCCCTTGACTGATCCCGACCTCAACTTGTATACTGATGGAAGTTCCTTTGTAGAAAAAGGACTTCGAAAAGCGGGGTATGCAGTGGTCAGTGATAATGGAA 6115
chr18_50449131-50449934    ----------------------------------------------------------------------------------------------------------------------------------------------------------- 260
AC241669.2_12558-20556     ----------------------------------------------------------------------------------------------------------------------------------------------------------- 5441
AC203657.2_c41170-33172    ----------------------------------------------------------------------------------------------------------------------------------------------------------- 5441
AC241258.1_c31008-23012    ----------------------------------------------------------------------------------------------------------------------------------------------------------- 5439
AC013759.6_36174-44171     ----------------------------------------------------------------------------------------------------------------------------------------------------------- 5440

Consensus_HERV-W           TACTTGAAAGTAATCCCCTCACTCCAGGAACTAGTGCTCAGCTGGCAGAACTAATAGCCCTCACTCGGGCACTAGAATTAGGAGAAGGAAAAAGGGTAAATATATATACAGACTCTAAGTATGCTTACCTAGTCCTCCATGCCCACGCAGCAATA 6270
chr18_50449131-50449934    ----------------------------------------------------------------------------------------------------------------------------------------------------------- 260
AC241669.2_12558-20556     ----------------------------------------------------------------------------------------------------------------------------------------------------------- 5441
AC203657.2_c41170-33172    ----------------------------------------------------------------------------------------------------------------------------------------------------------- 5441
AC241258.1_c31008-23012    ----------------------------------------------------------------------------------------------------------------------------------------------------------- 5439
AC013759.6_36174-44171     ----------------------------------------------------------------------------------------------------------------------------------------------------------- 5440

Consensus_HERV-W           TGGAGAGAAAGGGAATTCCTAACTTCCGAGGGAACACCTATCAAACATCAGGAAGCCATTAGGAGATTATTATTGGCTGTACAGAAACCTAAAGAGGTGGCAGTCTTACACTGCCGGGGTCATCAGAAAGGAAAGGAAAGGGAAATAGAAGGGAA 6425
chr18_50449131-50449934    ----------------------------------------------------------------------------------------------------------------------------------------------------------- 260
AC241669.2_12558-20556     ----------------------------------------------------------------------------------------------------------------------------------------------------------- 5441
AC203657.2_c41170-33172    ----------------------------------------------------------------------------------------------------------------------------------------------------------- 5441
AC241258.1_c31008-23012    ----------------------------------------------------------------------------------------------------------------------------------------------------------- 5439
AC013759.6_36174-44171     ----------------------------------------------------------------------------------------------------------------------------------------------------------- 5440

Consensus_HERV-W           CCGCCAAGCGGATATTGAAGCCAAAAGAGCCGCAAGGCAGGACCCTCCATTAGAAATGCTTATAGAAGGACCCCTAGTATGGGGTAATCCCCTCCGGGAAACCAAGCCCCAGTACTCAGCAGAAGAAATAGAATGGGGAACCTCACGAGGACATA 6580
chr18_50449131-50449934    ----------------------------------------------------------------------------------------------------------------------------------------------------------- 260
AC241669.2_12558-20556     ----------------------------------------------------------------------------------------------------------------------------------------------------------- 5441
AC203657.2_c41170-33172    ----------------------------------------------------------------------------------------------------------------------------------------------------------- 5441
AC241258.1_c31008-23012    ----------------------------------------------------------------------------------------------------------------------------------------------------------- 5439
AC013759.6_36174-44171     ----------------------------------------------------------------------------------------------------------------------------------------------------------- 5440

Consensus_HERV-W           GTTTCCTCCCCTCAGGATGGCTAGCCACCGAAGAAGGAAAAATACTTTTGCCTGCAGCTAACCAATGGAAATTACTTAAAACCCTTCACCAAACCTTTCACTTAGGCATTGATAGCACCCATCAGATGGCCAAATCATTATTTACTGGACCAGGC 6735
chr18_50449131-50449934    ----------------------------------------------------------------------------------------------------------------------------------------------------------- 260
AC241669.2_12558-20556     ----------------------------------------------------------------------------------------------------------------------------------------------------------- 5441
AC203657.2_c41170-33172    ----------------------------------------------------------------------------------------------------------------------------------------------------------- 5441
AC241258.1_c31008-23012    ----------------------------------------------------------------------------------------------------------------------------------------------------------- 5439
AC013759.6_36174-44171     ----------------------------------------------------------------------------------------------------------------------------------------------------------- 5440
```

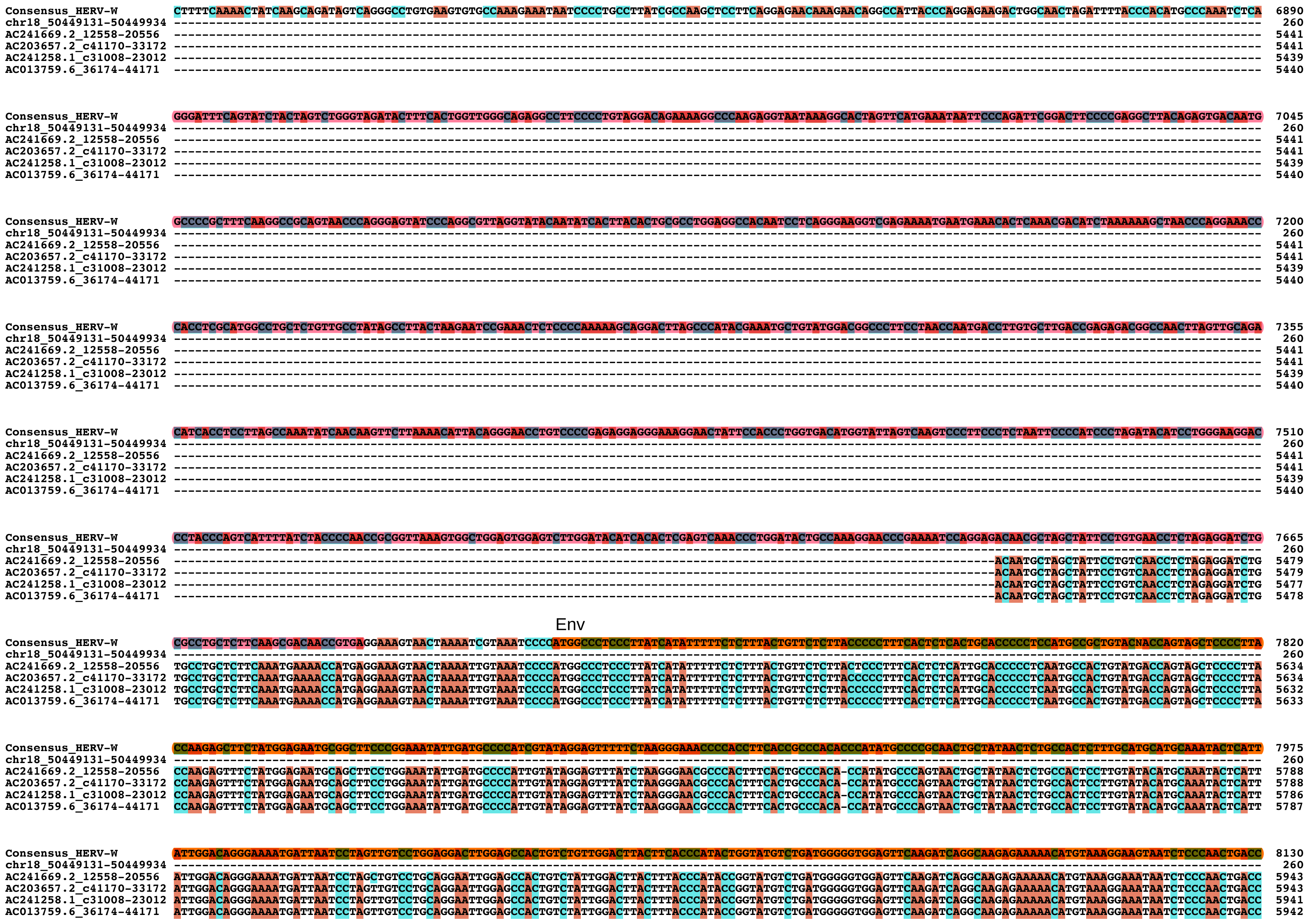
Env
Consensus_HERV-W
chr18_50449131-50449934
AC241669.2_12558-20556
AC203657.2_c41170-33172
AC241258.1_c31008-23012
AC013759.6_36174-44171

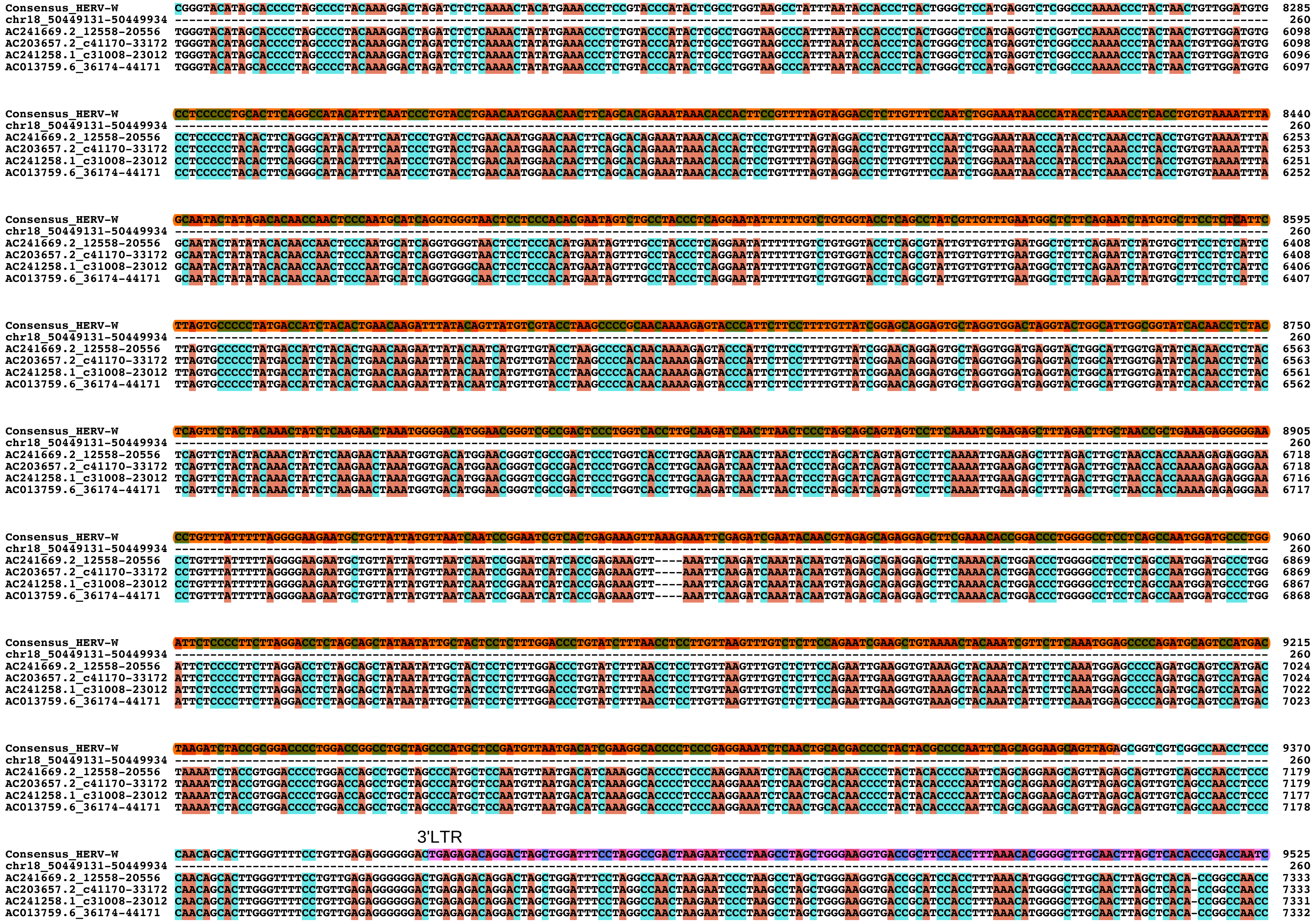
Consensus_HERV-W 8285
chr18_50449131-50449934 260
AC241669.2_12558-20556 6098
AC203657.2_c41170-33172 6098
AC241258.1_c31008-23012 6096
AC013759.6_36174-44171 6097
Consensus_HERV-W 8440
chr18_50449131-50449934 260
AC241669.2_12558-20556 6253
AC203657.2_c41170-33172 6253
AC241258.1_c31008-23012 6251
AC013759.6_36174-44171 6252
Consensus_HERV-W 8595
chr18_50449131-50449934 260
AC241669.2_12558-20556 6408
AC203657.2_c41170-33172 6408
AC241258.1_c31008-23012 6406
AC013759.6_36174-44171 6407
Consensus_HERV-W 8750
chr18_50449131-50449934 260
AC241669.2_12558-20556 6563
AC203657.2_c41170-33172 6563
AC241258.1_c31008-23012 6561
AC013759.6_36174-44171 6562
Consensus_HERV-W 8905
chr18_50449131-50449934 260
AC241669.2_12558-20556 6718
AC203657.2_c41170-33172 6718
AC241258.1_c31008-23012 6716
AC013759.6_36174-44171 6717
Consensus_HERV-W 9060
chr18_50449131-50449934 260
AC241669.2_12558-20556 6869
AC203657.2_c41170-33172 6869
AC241258.1_c31008-23012 6867
AC013759.6_36174-44171 6868
Consensus_HERV-W 9215
chr18_50449131-50449934 260
AC241669.2_12558-20556 7024
AC203657.2_c41170-33172 7024
AC241258.1_c31008-23012 7022
AC013759.6_36174-44171 7023
Consensus_HERV-W 9370
chr18_50449131-50449934 260
AC241669.2_12558-20556 7179
AC203657.2_c41170-33172 7179
AC241258.1_c31008-23012 7177
AC013759.6_36174-44171 7178
3'LTR
Consensus_HERV-W 9525
chr18_50449131-50449934 260
AC241669.2_12558-20556 7333
AC203657.2_c41170-33172 7333
AC241258.1_c31008-23012 7331
AC013759.6_36174-44171 7332

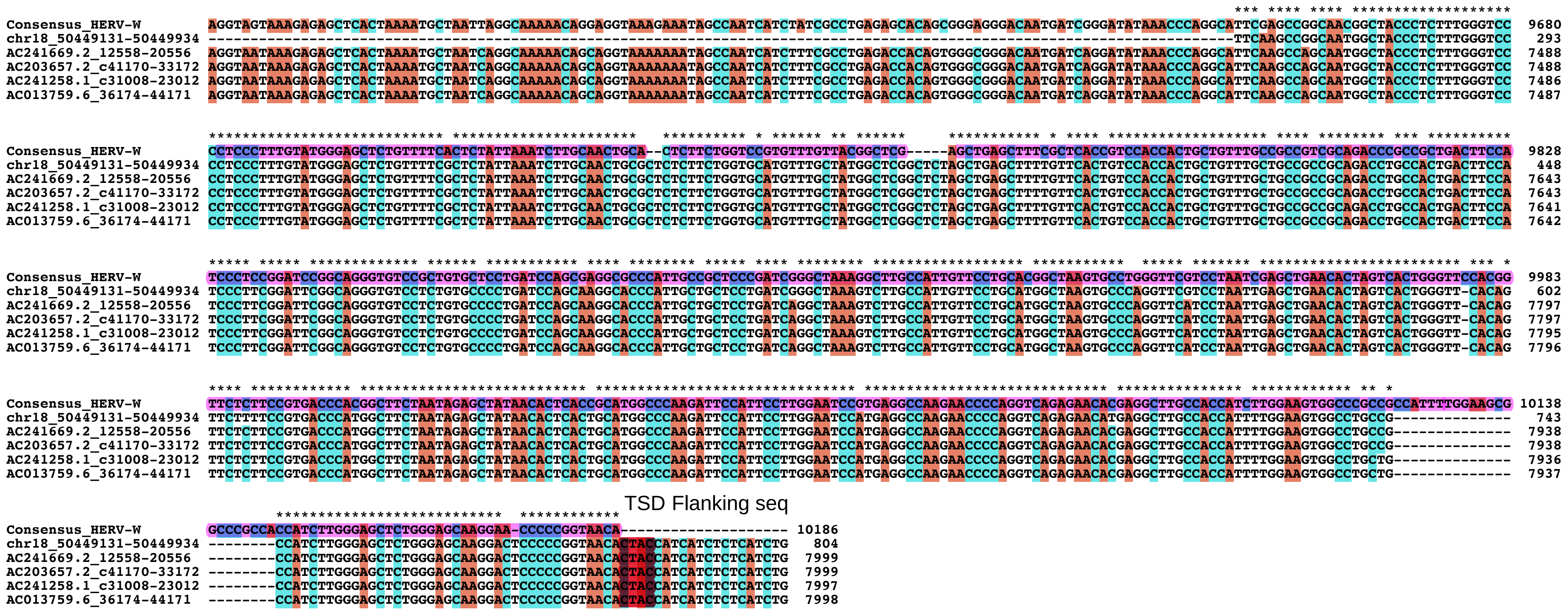


Additional file 5. Alignment of HERV-W consensus with 18q21.1_W2 solo LTR and provirus. The target site duplications (TSD) are marked in red. The 5' and 3'LTRs are highlighted in pink, and open reading frames for gag, pol and env genes are highlighted in green, light red and orange respectively. The HERV-W consensus sequence is from Repbase (LTR17 and HERV17).
