# Supplementary material for: Variation in proviral content among human genomes mediated by LTR recombination
Source: Mob DNA. 2018 Dec 18;9:36. doi: 10.1186/s13100-018-0142-3 (PMC6298018; doi:10.1186/s13100-018-0142-3)
Supplement: Supplementary file 6 — Alignment of 4q22.1_H8 solo LTR and provirus with HERV-H consensus. (PDF 410 kb) [file 13100_2018_142_MOESM6_ESM.pdf]

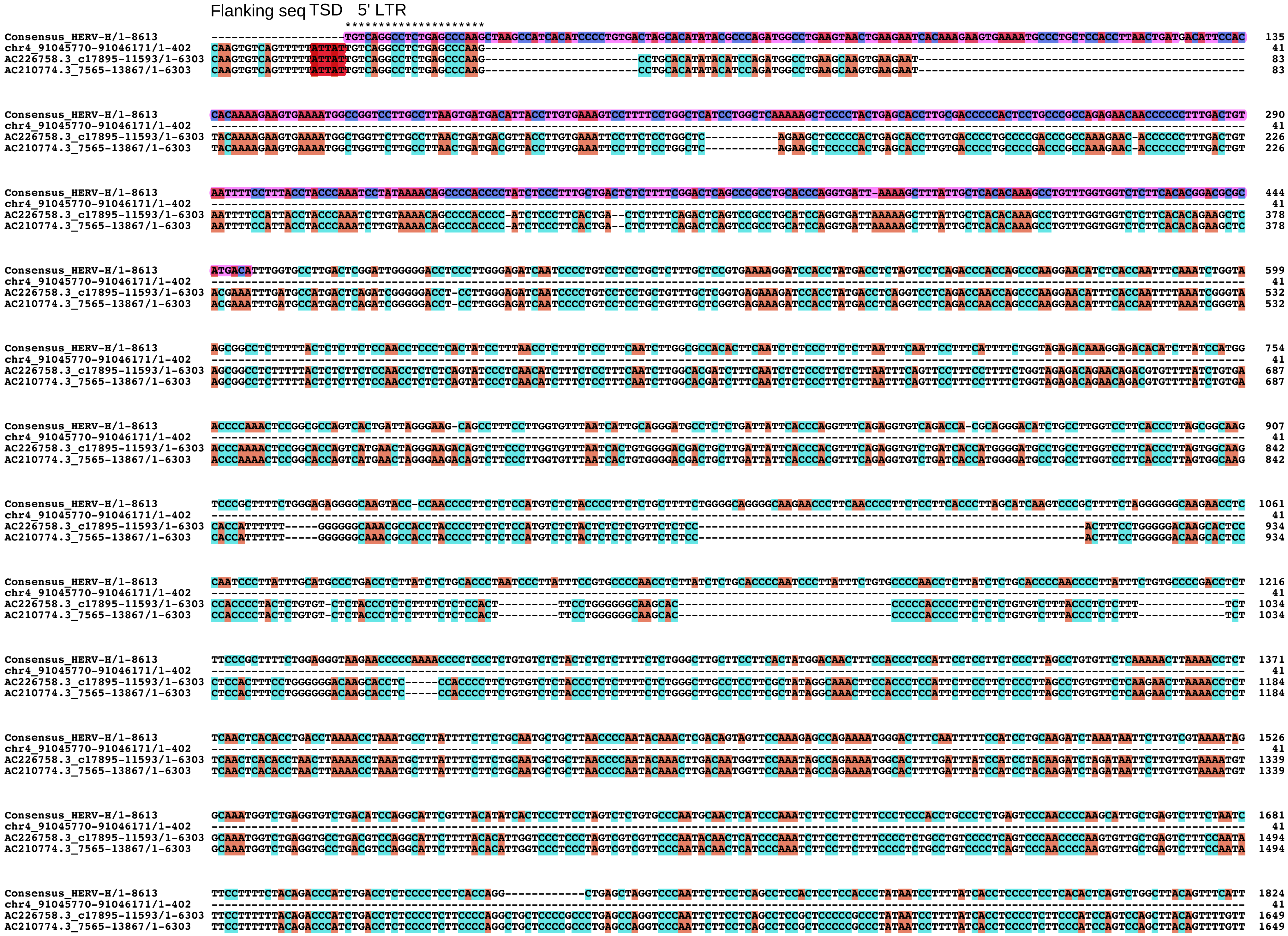
Flanking seq TSD 5' LTR
Consensus_HERV-H/1-8613
chr4_91045770-91046171/1-402
AC226758.3_c17895-11593/1-6303
AC210774.3_7565-13867/1-6303

```
Consensus_HERV-H/1-8613            CCGTGACTAGCCCTCCCCCACCTGCCCAGCAATTTCCTCTTAAAAAGTTGGCTGGAGCTAAAGACATAGTCAAGGTTAATGCTCCTTTTTCTTTATCTGACCTCTCCCAAATCAGTTAGCATTTAGACTCTTTTTCATCAAATACAAAAAACCCA  1979
chr4_91045770-91046171/1-402       -----------------------------------------------------------------------------------------------------------------------------------------------------------  41
AC226758.3_c17895-11593/1-6303     CTGCAACTAGCCCTCCCCCACCTGCCCAGCAATTTCCTCTTTAAAAGGTGGCTGGAGCTAAAGGCATAGTCAAGGTTACTGCTCCTTTTTCTTTAACCAACCTCTCCCAAATCAGTTAGCATGTAGGCTCTTTTTCATCAAATAT-AAAAACCCA  1803
AC210774.3_7565-13867/1-6303       CTGCAACTAGCCCTCCCCCACCTGCCCAGCAATTTCCTCTTTAAAAGGTGGCTGGAGCTAAAGGCATAGTCAAGGTTACTGCTCCTTTTTCTTTAACCAACCTCTCCCAAATCAGTTAGCATGTAGGCTCTTTTTCATCAAATAT-AAAAACCCA  1803

Consensus_HERV-H/1-8613            GCCCAGTTCATGGCTCATTCGGCAGCAACCCTGAGACGCTTTACAGCCTTAGACCCTAAAAGGTCAAAAGGCCGTCTTATCCTCAATATACATTTTATTACCCAATCTGCTCCAAACATTAAATAAAACTCCAAAAATTAAATTCTGGCCCTCAA  2134
chr4_91045770-91046171/1-402       -----------------------------------------------------------------------------------------------------------------------------------------------------------  41
AC226758.3_c17895-11593/1-6303     GCCCAGTTCATGGCTCATTTGGCAGCAACCCTGAGACGCTTTACGGCCCTAGGCCCTAAAAGGTCAAAAGGCCATCTTATTCTCAATATACATTTTATTACCCAATCTACTCCCAACATTAAATAAAATTCCAAAAATTAAATTCCCACCCTCAA  1958
AC210774.3_7565-13867/1-6303       GCCCAGTTCATGGCTCATTTGGCAGCAACCCTGAGACGCTTTACGGCCCTAGGCCCTAAAAGGTCAAAAGGCCATCTTATTCTCAATATACATTTTATTACCCAATCTACTCCCAACATTAAATAAAATTCCAAAAATTAAATTCCCACCCTCAA  1958

Consensus_HERV-H/1-8613            ACCCCACAACAGGATTTAATTAACCTCACCTTCAAGGTGTACAATAATAGAAAA--------------------------AAGTTGCAATTCCTTGCCTCCACTGTGAGACAAACCCCAGACACATCTCCAGCACACAAGAACTTCGAAATGCC  2262
chr4_91045770-91046171/1-402       -----------------------------------------------------------------------------------------------------------------------------------------------------------  41
AC226758.3_c17895-11593/1-6303     ACCCCACAATAGGACTTAATTAACCTCAACTTCAAGTTGTATAATAGTAGAGTAGAGGCAGCCAAGTAGCAAGGTATTTCTGAGTTGCAGTTCCTTGCCTCCACTGTGAGAGAAACCCCAGCCACATCTCCAGCACACAAGAACTTCAAAATGCC  2113
AC210774.3_7565-13867/1-6303       ACCCCACAATAGGACTTAATTAACCTCAACTTCAAGTTGTATAATAGTAGAGTAGAGGCAGCCAAGTAGCAAGGTATTTCTGAGTTGCAGTTCCTTGCCTCCACTGTGAGAGAAACCCCAGCCACATCTCCAGCACACAAGAACTTCAAAATGCC  2113

Consensus_HERV-H/1-8613            TCAACCTCAGGTGCCAGGGGTTCCTCCAGAACCTTCTCCCCCAGGAGCTTGCTACAAGTGCCAGAAATCTGGCCACTGGGCCAAGGAATGCCCACAGACCAGGATTCCTCCTAAGCTGTATCCCATCTCTGTGGGACCCCACTAAAAATCAGACT  2417
chr4_91045770-91046171/1-402       -----------------------------------------------------------------------------------------------------------------------------------------------------------  41
AC226758.3_c17895-11593/1-6303     TAAGCCACAGTGGTGAGGCATTCCTTCAGGACCTCCTCCCCCAGGATCTTGCTTCAAGTGCCGGAAATCTGGCCACCAGGCCAAGGAATGCCTGCAGCCTGGGATTCCTCCTAAGCTGTGTCCCAGCTGTGTGGGTCCCCACTGGAAATTCAACT  2268
AC210774.3_7565-13867/1-6303       TAAGCCACAGTGGTGAGGCATTCCTTCAGGACCTCCTCCCCCAGGATCTTGCTTCAAGTGCCGGAAATCTGGCCACCAGGCCAAGGAATGCCTGCAGCCTGGGATTCCTCCTAAGCTGTGTCCCAGCTGTGTGGGTCCCCACTGGAAATTCAACT  2268

Consensus_HERV-H/1-8613            GTTCAACTCACCTGGCAGCCACTTCCAGAGCCCCTGGAACTCTAGCCCAAGGCTCTCTGACTGACCCCTTCTGAGATCTTCTTGGCTTAGCAGCTGAAGACTGACACTGCCAGATCGCCTCGGAAGCCTACAGGACCATCACAGATGCTCCAGGT  2572
chr4_91045770-91046171/1-402       -----------------------------------------------------------------------------------------------------------------------------------------------------------  41
AC226758.3_c17895-11593/1-6303     GTCCAA-----------------------------------CTAGCCCAAGG--CTCTGACTTACTTCTTCCCAGATCTTCTCAGCTTAGTGGCTGAAGACTGGCGCTGCCTGTGTGCCTTGGAAGCCTCCTGGACCATCACAGATGCTTTGGGT  2386
AC210774.3_7565-13867/1-6303       GTCCAA-----------------------------------CTAGCCCAAGG--CTCTGACTTACTTCTTCCCAGATCTTCTCAGCTTAGTGGCTGAAGACTGGCGCTGCCTGTGTGCCTTGGAAGCCTCCTGGACCATCACAGATGCTTTGGGT  2386

Consensus_HERV-H/1-8613            AACTCTCACAGTAGAGGGTAAGTCTGTCCCCTTCTTAATCAATATGGAGGCTACCCACTGCACATTACCTTCTTTTCAAGGGCCTGTTTCCTTTGCCTCCATAACTGTTGTGGGTATTGACGGCCAGGCTTCTAAACCTCTTAAAACTCCCCAAC  2727
chr4_91045770-91046171/1-402       -----------------------------------------------------------------------------------------------------------------------------------------------------------  41
AC226758.3_c17895-11593/1-6303     AACTCTTACAGTGGAGGGTAAGTCCATCCCCTTCTTAATCAATACAGAGGCTACCCACTCCACATTACCTTCTTTTCAAGGGCCTGTTTCCCTTGCCTCCATAACTGTTGTGCGTATTGATGGCCAGGCTTCTAAACCTCTTCAAACTCCCCAAC  2541
AC210774.3_7565-13867/1-6303       AACTCTTACAGTGGAGGGTAAGTCCATCCCCTTCTTAATCAATACAGAGGCTACCCACTCCACATTACCTTCTTTTCAAGGGCCTGTTTCCCTTGCCTCCATAACTGTTGTGCGTATTGATGGCCAGGCTTCTAAACCTCTTCAAACTCCCCAAC  2541

Consensus_HERV-H/1-8613            TCTAGTACCAACTTAGACAATACTCTTTTAAGCACTCCTTTTTAGTTATCCCCACTTGCCCAGTTCCCTTATGAGGCCGAGACACTTCAACTAAATTATCTGCTTCCCTGACTATTCCTGGACTACAGCTACATCTCATTGCTGCCCTTCTTCCC  2882
chr4_91045770-91046171/1-402       -----------------------------------------------------------------------------------------------------------------------------------------------------------  41
AC226758.3_c17895-11593/1-6303     TCTGGTGCCAACTTGGACAGTATTCTTTTATGCACTCCTTTTTAGTTATCCCCACCTGCCCAGTTCCCTTATTAGGCCAAGATATTTTAACTAAATTATCTGCTTCCCTGACCATTCCTAGGCTACAGCCACACCTCATTGCCACCCATTTCCCC  2696
AC210774.3_7565-13867/1-6303       TCTGGTGCCAACTTGGACAGTATTCTTTTATGCACTCCTTTTTAGTTATCCCCACCTGCCCAGTTCCCTTATTAGGCCAAGATATTTTAACTAAATTATCTGCTTCCCTGACCATTCCTAGGCTACAGCCACACCTCATTGCCACCCATTTCCCC  2696

Consensus_HERV-H/1-8613            AATCCAAAGCCTCCTTTGCATCTT---CTTGTATCCCCCAACCTTAACCCACAAGTATAAGATACCTCTATTCCCTCCTTGGTGACCAATCATGCACCCCTTACCATCTCATTAAAACCTAATCACTCTTACCCGGCTCAATGCCAAGATCCCAT  3034
chr4_91045770-91046171/1-402       -----------------------------------------------------------------------------------------------------------------------------------------------------------  41
AC226758.3_c17895-11593/1-6303     AGTTCAAAGCCTCCTTCACATCCTCCCCTTGTATCTCCCCACCTTAATCCACAAGTATAGGACATCTCTACTCCATCCTTGGCGACCAATCATGCACCCCTTACCATCCCATTAAAACCTAATCACCCTTACCCCACTCAACGCCAATATCCCAT  2851
AC210774.3_7565-13867/1-6303       AGTTCAAAGCCTCCTTCACATCCTCCCCTTGTATCTCCCCACCTTAATCCACAAGTATAGGACATCTCTACTCCATCCTTGGCGACCAATCATGCACCCCTTACCATCCCATTAAAACCTAATCACCCTTACCCCACTCAACGCCAATATCCCAT  2851

Consensus_HERV-H/1-8613            CCCACAGCATGCTTTAAAAGGATTAAAACCTGTTATCACTCGCCTGCTAGAGCATGGCCTTTTAAAGCCTATAAACTCTCCTTACAATTCCCCCATTTTACCTGTCCTAGAACCAGACAAGCCTTACAG----GTTCAGGATCTGTGTCTTATCA  3185
chr4_91045770-91046171/1-402       -----------------------------------------------------------------------------------------------------------------------------------------------------------  41
AC226758.3_c17895-11593/1-6303     CCCACAGCAGGCTGTAAAAGGAATAAAGCCTGTTATCACTTGCCTGTTACAGCATGGCCTTTTAAAGCCTATAAACTCTCCTTAC-ATTCCCCCATTTTACCTGTCCAAAAACGAGACAAGTCTTACAGGTTAGTTCAGGATCTGCACCTTATCA  3005
AC210774.3_7565-13867/1-6303       CCCACAGCAGGCTGTAAAAGGAATAAAGCCTGTTATCACTTGCCTGTTACAGCATGGCCTTTTAAAGCCTATAAACTCTCCTTAC-ATTCCCCCATTTTACCTGTCCAAAAACGAGACAAGTCTTACAGGTTAGTTCAGGATCTGCACCTTATCA  3005

Consensus_HERV-H/1-8613            ATGAAATTGTTTTCCCTATCCACCCTGTGGTGCTGAACCCATATACTCTCCTATCCTCAATACCTC-----------CCTCTACAACCCATTATTCTGTTCTAGATCTCAAACATGCTTTCTTTACTATCCCTTTACACCCTTCAACCCAGCCTC  3329
chr4_91045770-91046171/1-402       -----------------------------------------------------------------------------------------------------------------------------------------------------------  41
AC226758.3_c17895-11593/1-6303     ACCAAATTGTTTTGCCTATCCACCCCATGGTGCCAAACCCATATACTCTCCTATCCTCGATACCTCCCCCGACAACACCTCCACAACCCATTATTCTGTTCTGGATCTCAAACATGCTTTCTTTACTATTCCTTTGCAGCCTTCATCCCAGCCTG  3160
AC210774.3_7565-13867/1-6303       ACCAAATTGTTTTGCCTATCCACCCCATGGTGCCAAACCCATATACTCTCCTATCCTCGATACCTCCCCCGACAACACCTCCACAACCCATTATTCTGTTCTGGATCTCAAACATGCTTTCTTTACTATTCCTTTGCAGCCTTCATCCCAGCCTG  3160

Consensus_HERV-H/1-8613            TCTTCGTTTTCACCTGGACTGACCCTGACACCCATCAGTCCCAGCAGCTTACCTGGGCTGTAATGCTGCAAGGTTTCAGGGGCAGCCCTTATTATTTCAGCCAAGCTCTTTCTCATGATTTACTTTCTTTCCACCCCTCCACTTCTCACCTTATT  3484
chr4_91045770-91046171/1-402       -----------------------------------------------------------------------------------------------------------------------------------------------------------  41
AC226758.3_c17895-11593/1-6303     TCTTCACTTTCACTTGGACTGACCCTGATACCCATCAGCCTCAGCAAATTACCTGGGTTGTACTGCCACAAGGCTTCACGGACAGCCCCCATTACTTCAGTC-----------------------------------------------------  3262
AC210774.3_7565-13867/1-6303       TCTTCACTTTCACTTGGACTGACCCTGATACCCATCAGCCTCAGCAAATTACCTGGGTTGTACTGCCACAAGGCTTCACGGACAGCCCCCATTACTTCAGTC-----------------------------------------------------  3262

Consensus_HERV-H/1-8613            CAATATATTGGTGATGTTCTTCTTTGTAGCCCCTCCTTTGAATCTTCTCAACAAGACACACTTCTGCTCCTTCAGCATTTATTCTCCAAAGGATATCCCCCTCCAAAGCTCAAATGTCTTCTCCATCCGTTACCTACCTTGGCATAATTCTTCAT  3639
chr4_91045770-91046171/1-402       -----------------------------------------------------------------------------------------------------------------------------------------------------------  41
AC226758.3_c17895-11593/1-6303     ---------------------------------------------------------------------------------------------------------AAGCTCAAATTTCTTCCTCATCCATTACCTATCTTGGCATAATTCTTCAT  3312
AC210774.3_7565-13867/1-6303       ---------------------------------------------------------------------------------------------------------AAGCTCAAATTTCTTCCTCATCCATTACCTATCTTGGCATAATTCTTCAT  3312
```

```
Consensus_HERV-H/1-8613              AAAAACACACGTGCCCTCCCTGCTGATAGTGTCTGACTGATCTCTCAAACCCCAACCCCTTCTACAAAACAACAACTCTTTTCCATCCTAGGCATGGTTGGATACTTTCGTGTTAGGATACCTGGTTTTGCCATCCTAACAAAACCATTATATAA  3794
chr4_91045770-91046171/1-402         -----------------------------------------------------------------------------------------------------------------------------------------------------------   41
AC226758.3_c17895-11593/1-6303       GAAAACACATGTGCTCTGCCTCCTGATCGTGTCTGACTGATATCTCAAACCCCAATACCTTCTACAAAACAACAACTC-TTTCCTTCCTAGTCATGGTTAGGTACTTCCACCTTTGGATACCTAGTTTTACCATCCTAACAAAACCATTATATAA  3466
AC210774.3_7565-13867/1-6303         GAAAACACATGTGCTCTGCCTCCTGATCGTGTCTGACTGATATCTCAAACCCCAATACCTTCTACAAAACAACAACTC-TTTCCTTCCTAGTCATGGTTAGGTACTTCCACCTTTGGATACCTAGTTTTACCATCCTAACAAAACCATTATATAA  3466

Consensus_HERV-H/1-8613              ACTCACAAAAGGAAACCTAGTTGACCCCATAGATCCTAAATCGTTTCCCCACTCCTCTTTCCATTCCTTGAAGACAGCTTTAGAGACTGTCTCCACTCTAGCTCTCCCTGACTCATCCCAACACTTTTCATTACACACAGCTGAAGTGCAGGGCT  3949
chr4_91045770-91046171/1-402         -----------------------------------------------------------------------------------------------------------------------------------------------------------   41
AC226758.3_c17895-11593/1-6303       ACTCACAAAAGGAAACCTAGCTGACCCCATAGATCCTAAATCCTTT-CCCACTCCTTTTTCTGTTCCTTGAAGACAGCTTTAGAGACTGCCCCCACACTATCTCTCCCTGACTCATCCCAACCCTTTTCATTACACACAGCCAAAGTACAGGACT  3620
AC210774.3_7565-13867/1-6303         ACTCACAAAAGGAAACCTAGCTGACCCCATAGATCCTAAATCCTTT-CCCACTCCTTTTTCTGTTCCTTGAAGACAGCTTTAGAGACTGCCCCCACACTATCTCTCCCTGACTCATCCCAACCCTTTTCATTACACACAGCCAAAGTACAGGACT  3620

Consensus_HERV-H/1-8613              GTGCAGTCAGAATTCTTACACAAGGACCGGGATCGCATCCTGTAGCCTTTTTGTCCAAACAACTTGACCTTACTGTTTTAGGCTGGCCATCATGTCTCCATGCAGCGTCTGCTGCCACCCTAATACTTTTAGAGGCCCTCAAAATCACAAACTAT  4104
chr4_91045770-91046171/1-402         -----------------------------------------------------------------------------------------------------------------------------------------------------------   41
AC226758.3_c17895-11593/1-6303       GTGCAGTCAGAATTCTTACACAAGGACTGGGACTGCACCCTGTAGCCTTTTTGTCCAAACAACTTGACCTTACTGTTTTAGGCTGGCCATCATGTATCCGTGCAGCGGCTGCCGCCATCCTAATACTTTGAGAGGCCCTCAAACTCACAAACTAT  3775
AC210774.3_7565-13867/1-6303         GTGCAGTCAGAATTCTTACACAAGGACTGGGACTGCACCCTGTAGCCTTTTTGTCCAAACAACTTGACCTTACTGTTTTAGGCTGGCCATCATGTATCCGTGCAGCGGCTGCCGCCATCCTAATACTTTGAGAGGCCCTCAAACTCACAAACTAT  3775

Consensus_HERV-H/1-8613              GCTCAACTCATTCTCTACAGCTCTCATAATTTCCAAAATCTATTTTCTTCCTCACACCTGACACATATACTTTCTGCTCCCCGGCTCCTTCAGATATACTCACTC--CATTTATTCTCCCACAATTACCATTATTCCTGGCCTGGACTTCAATCC  4257
chr4_91045770-91046171/1-402         -----------------------------------------------------------------------------------------------------------------------------------------------------------   41
AC226758.3_c17895-11593/1-6303       GCTCAACTCACTCTCTACAGCTCTCATAACTTCCAAAATTTATTTTCTTCCTCACATCTGACACATATACTTTCTGCTCCCTGGCTCCTTCAGCTATACTCACTCTTTGTTGAGTCTCCCACAGGTATCATT-------GGCCGGACTTCAATCC  3923
AC210774.3_7565-13867/1-6303         GCTCAACTCACTCTCTACAGCTCTCATAACTTCCAAAATTTATTTTCTTCCTCACATCTGACACATATACTTTCTGCTCCCTGGCTCCTTCAGCTATACTCACTCTTTGTTGAGTCTCCCACAGGTATCATT-------GGCCGGACTTCAATCC  3923

Consensus_HERV-H/1-8613              GGCCTCCCACATTATTCTGGATACCATACCTGACCCTCATGACTGCATCTCTCTGATCCACCTGACGTTCACCCCATTTCCCCACATTTCCTTCTGCCCTGTTTCTCACCCTGATCACACTTGGTTTATTGATGGCAGTTCCACCAGGCCTAATC  4412
chr4_91045770-91046171/1-402         -----------------------------------------------------------------------------------------------------------------------------------------------------------   41
AC226758.3_c17895-11593/1-6303       GGCCTCCGACATTATTCCGGATACCACACCTGACCCCCAAGACTGTATCTCTCTGATCCACCTGACATTTGCCCCATTTCCCCATATTTCCTTCTTTCCTGTTCCTCACCCTGATTACATTTGGTTTACTGATGGCAGTTCCACCAGGCCTAATC  4078
AC210774.3_7565-13867/1-6303         GGCCTCCGACATTATTCCGGATACCACACCTGACCCCCAAGACTGTATCTCTCTGATCCACCTGACATTTGCCCCATTTCCCCATATTTCCTTCTTTCCTGTTCCTCACCCTGATTACATTTGGTTTACTGATGGCAGTTCCACCAGGCCTAATC  4078

Consensus_HERV-H/1-8613              GCCACTCACCAGCAAAGGCAGGATATGCTA------------------------------------------------------------------TGAACTAGTTGCCTTAATTCAAGCCCTCACTCTTGCAAAAGGACTACGTGTCAATATCT  4501
chr4_91045770-91046171/1-402         -----------------------------------------------------------------------------------------------------------------------------------------------------------   41
AC226758.3_c17895-11593/1-6303       ACCACTCACCAGCAAAGGCAGGCTATGCTATATAGTATCTTCCACATCTATAATTGAGGCTACTGCTCTGCCCCGCTCCACTACCTCTCAGCAAGCTGAACTCATTGCCTTAACTCGGGACCTCACTCTTGCAAAGGGACTACACATCAATACTT  4233
AC210774.3_7565-13867/1-6303         ACCACTCACCAGCAAAGGCAGGCTATGCTATATAGTATCTTCCACATCTATAATTGAGGCTACTGCTCTGCCCCGCTCCACTACCTCTCAGCAAGCTGAACTCATTGCCTTAACTCGGGACCTCACTCTTGCAAAGGGACTACACATCAATACTT  4233

Consensus_HERV-H/1-8613              ATACTGATTCTAAATATGCCTTTCATATTCTGCACCACCATGCGGTCATATGGGCTGAAAGAGGTTTCCTCACTACAC-AAGTGTCCTCCATCATTAATGCCTCTTTAA-GAAAACTCTGCTCAAGGCTGCTTTACTTCCAAAGGAAGCTGGGGT  4654
chr4_91045770-91046171/1-402         -----------------------------------------------------------------------------------------------------------------------------------------------------------   41
AC226758.3_c17895-11593/1-6303       ATACTGACTCTAAATATGCCTTCCATATCCTGCACCACCATGCTGTTACATGGGCAGAAAGAGGTTTCCTTACTACACAAAAGGTCCTCCATCATTAATGCCTCTTTAATAAAAACTCTTCTCAAGGCCTCTTTGCTTCCAAAGGAAGCTGGAGT  4388
AC210774.3_7565-13867/1-6303         ATACTGACTCTAAATATGCCTTCCATATCCTGCACCACCATGCTGTTACATGGGCAGAAAGAGGTTTCCTTACTACACAAAAGGTCCTCCATCATTAATGCCTCTTTAATAAAAACTCTTCTCAAGGCCTCTTTGCTTCCAAAGGAAGCTGGAGT  4388

Consensus_HERV-H/1-8613              CATTCACTGCAAGGGGCATCAAAAGACTTCAGATCCCATTGCTCTAGGCAATGCTTATGCTGATAAGGTGGCTAGACAAGCAGCTAGCTCTCCAACTTTTGTCCCTCATGGCCAGTTTTTCTCCTTCACATCCG----TCACTCCCACCTACTCC  4805
chr4_91045770-91046171/1-402         -----------------------------------------------------------------------------------------------------------------------------------------------------------   41
AC226758.3_c17895-11593/1-6303       CATTCACTGCAAGGGCCATCAAAAGGCGTCATATCCCATTGCTCTAGGCAGTGCTTATGCTGATAACGTAGCTAAAGAAGCAGCTAACATTCCAACTTCTGTCCCTCACGGCCAGTTTTTCTTCTTCTCATCCATCGTTCACTCTCACCTACTCC  4543
AC210774.3_7565-13867/1-6303         CATTCACTGCAAGGGCCATCAAAAGGCGTCATATCCCATTGCTCTAGGCAGTGCTTATGCTGATAACGTAGCTAAAGAAGCAGCTAACATTCCAACTTCTGTCCCTCACGGCCAGTTTTTCTTCTTCTCATCCATCGTTCACTCTCACCTACTCC  4543

Consensus_HERV-H/1-8613              ACAGCTGAAACTTCCACCTATCAAGCTCTTCCCCCGCAAGGTAAATGGTTCTTAGACCAAGGAAAATATCTCCTTCCAGCCTCACAGGCCCATTCTATTCTGTCGTCATTTCATAACCTTTTCCATGTAGGTTACAAGCCACTAGCCTGTCTCTT  4960
chr4_91045770-91046171/1-402         -----------------------------------------------------------------------------------------------------------------------------------------------------------   41
AC226758.3_c17895-11593/1-6303       CCAGCTGAAACTGCCACCTATCAATCTCTTCCCACACAAGGCAAATGGTTCTTGGACCAAGGAAAATATCTCCTTCCGGCCTCACAGGCCTATTGCATTGTGTCGTCATTTCATAAACTCTTCCATGTAAGTTACAAGCCACTAGCCCATCTCTT  4698
AC210774.3_7565-13867/1-6303         CCAGCTGAAACTGCCACCTATCAATCTCTTCCCACACAAGGCAAATGGTTCTTGGACCAAGGAAAATATCTCCTTCCGGCCTCACAGGCCTATTGCATTGTGTCGTCATTTCATAAACTCTTCCATGTAAGTTACAAGCCACTAGCCCATCTCTT  4698

Consensus_HERV-H/1-8613              AGGACCTCTCATTTCCTTTCCATCATGGAAATCTATCCTCAAGGAGATCACTTCTCAGTGTTCCATCTGCTATTCTGCTACCCCTCAGGGATTGTTCAGG-CCTCCTCCCTTTCCTACACATAAAGCTCGGGGATTTGCCCCTGCCCAGGACTGG  5114
chr4_91045770-91046171/1-402         -----------------------------------------------------------------------------------------------------------------------------------------------------------   41
AC226758.3_c17895-11593/1-6303       AGAACCTCTCATTTCCTTTCCATCGTGGAAATCTATCCTTAAGGAAATCACTTCTCAGTATTCCATCTGCTATTCTACTACTCCTCAGGAATTTCTCAGGCCCCCCTCCCTTCCCTACACATCAAGCTTGGGGATTTGCTTCTGCCCAGGACTGG  4853
AC210774.3_7565-13867/1-6303         AGAACCTCTCATTTCCTTTCCATCGTGGAAATCTATCCTTAAGGAAATCACTTCTCAGTATTCCATCTGCTATTCTACTACTCCTCAGGAATTTCTCAGGCCCCCCTCCCTTCCCTACACATCAAGCTTGGGGATTTGCTTCTGCCCAGGACTGG  4853

Consensus_HERV-H/1-8613              CAAATTGACTTTACTCACATGCCTCGGGTCAGAAAACTAAAATATCTCTTAGTCTGGGTAGACACTTTCACTGGGTGGGTAGAGGCCTTTCCCATAGAGTCTGAGAAGGCCACCGCGGTCATTTCTTCCCTTCTGTCAGACATAATTCCTTGGTT  5269
chr4_91045770-91046171/1-402         -----------------------------------------------------------------------------------------------------------------------------------------------------------   41
AC226758.3_c17895-11593/1-6303       CAAATTGACTTTACTCACATGCCCCGAGTCAGGAAACTAAAATACCTCTTGGTCTGGGTAGACACTTTCACTGGATGGGTAGAGACCTTTCCTGCAGGGTCTGAGAAGGCCACTGCGATCATTTATTCCCTTCTGTCAGACATAA----------  4998
AC210774.3_7565-13867/1-6303         CAAATTGACTTTACTCACATGCCCCGAGTCAGGAAACTAAAATACCTCTTGGTCTGGGTAGACACTTTCACTGGATGGGTAGAGACCTTTCCTGCAGGGTCTGAGAAGGCCACTGCGATCATTTATTCCCTTCTGTCAGACATAA----------  4998

Consensus_HERV-H/1-8613              TGGCCTTCCCTTCTCTATACAGTCTGATAACGGACCAGCCTTTACTAGTTAAATCACCCAAGCAGTTTCTCAGGCTCTTGGTATTCAGTGGAACCTTCATATCCCTTAACATCCTCAATCTTCAGGAAAGGTAAAACCGACTAATGGTCTTTTAA  5424
chr4_91045770-91046171/1-402         -----------------------------------------------------------------------------------------------------------------------------------------------------------   41
AC226758.3_c17895-11593/1-6303       -----TTCCCACCTCTATACAGTCTAATAACAGACCGGCCTTTACTATTCAAATCACCCAAGCAGTTTCTCAGGCTCTTGATATTCAATGAAACCTTCATACCCCTTACTGTCCTCAATCTTCAAAAAAGGTAGAACAGACAAATGGTCTTTTAA  5148
AC210774.3_7565-13867/1-6303         -----TTCCCACCTCTATACAGTCTAATAACAGACCGGCCTTTACTATTCAAATCACCCAAGCAGTTTCTCAGGCTCTTGATATTCAATGAAACCTTCATACCCCTTACTGTCCTCAATCTTCAAAAAAGGTAGAACAGACAAATGGTCTTTTAA  5148
```

```
Consensus_HERV-H/1-8613              AGACACACCTCACCAAGCTCAGCCTCCAACTTAAAAAGGATTGGACAGTACTTTTACCTCTCGCCCTTCTCAGAATTAGAACCTGTCCTCGAGATGCTACAGGGTACAGTCCATTTGAACTTTTATATGGACGCACTTTCTTGCTTGGTCCCAAC  5579
chr4_91045770-91046171/1-402         -----------------------------------------------------------------------------------------------------------------------------------------------------------    41
AC226758.3_c17895-11593/1-6303       AAACACACCTCACCAAGCTCAGCCACAAACTTAAAAAGGACTGGACAATACTTTTACCACTTTCCCTTCTCAGAATTCGGGCCTGTCCTCAGAATGCTACAGGCTACAGCCCATTTGAGCTCCTGTATGGACACTCCTTTTTACTAGGCCCCAGC  5303
AC210774.3_7565-13867/1-6303         AAACACACCTCACCAAGCTCAGCCACAAACTTAAAAAGGACTGGACAATACTTTTACCACTTTCCCTTCTCAGAATTCGGGCCTGTCCTCAGAATGCTACAGGCTACAGCCCATTTGAGCTCCTGTATGGACACTCCTTTTTACTAGGCCCCAGC  5303

Consensus_HERV-H/1-8613              CTCATCCCAGACACCAGCCCTCTAGGCGACTATCTTCCAGTCCTCCAACAGGCTAGGCAGGAAAGTCACCAGGCTGCTAATCTTCTCTTGCCTACTCCAGATCCCCAGCCATATGAAGACACTCTAGCTGGACGATCAGTTCTTGTTAAGAATCT  5734
chr4_91045770-91046171/1-402         -----------------------------------------------------------------------------------------------------------------------------------------------------------    41
AC226758.3_c17895-11593/1-6303       CTCATTCCAGACACCAGCC----------------------------------------------------------------------------------------------------------------------------------------  5322
AC210774.3_7565-13867/1-6303         CTCATTCCAGACACCAGCC----------------------------------------------------------------------------------------------------------------------------------------  5322

Consensus_HERV-H/1-8613              GACCCCTCAAACTCTACAACCTCGATGGACTGGACCCTACTTAGTCATCTATAGTACCCTGACTGCCGTCCGCCTGCAGGATCCTCCCCACTGGGTTCACCATTCCAGAATAAAGCTGTGTCCATTGGACAGCCAGCCTAATCCCTCCTCTTCCT  5889
chr4_91045770-91046171/1-402         -----------------------------------------------------------------------------------------------------------------------------------------------------------    41
AC226758.3_c17895-11593/1-6303       -----------------------------------------------------------------------------------------------------------------------------------------------------------  5322
AC210774.3_7565-13867/1-6303         -----------------------------------------------------------------------------------------------------------------------------------------------------------  5322

Consensus_HERV-H/1-8613              CCTGGAAGTCGCAATTACTCTCCCCTACTTCCCTTAAACTCACTCGTATTTCTGAAGAACAGTAATAACCCTTATGAGCCTAATACATCCCTTCATTCTATTAGGTCTGTTTGTCCTTACCCTACTTTTTGCAACAGGGCTTTATGAACTCACCC  6044
chr4_91045770-91046171/1-402         -----------------------------------------------------------------------------------------------------------------------------------------------------------    41
AC226758.3_c17895-11593/1-6303       -----------------------------------------------------------------------------------------------------------------------------------------------------------  5322
AC210774.3_7565-13867/1-6303         -----------------------------------------------------------------------------------------------------------------------------------------------------------  5322

Consensus_HERV-H/1-8613              CCACCACTTAGGCTGAGCCCAAAAAATCTTGTCATCCCTACTATTTTCTGTCCAGTCATACTCCTATTCTCTGCTCTCAACTACTTATAAATGCCGTACTCTT-------------GTTTACACTGCTGGTTTACACTGTTTCTTCAAGCCATCA  6186
chr4_91045770-91046171/1-402         -----------------------------------------------------------------------------------------------------------------------------------------------------------    41
AC226758.3_c17895-11593/1-6303       ---CAACTTGGACAGTGCCCCAAAAA-CCTGTCATCCCTACTATCTTCTGTCTAGTCATACTCCCATTCACCGTTCTCAATTACTCATAAATGCCCTGCTCTTGTTTACACTGCTGGTTTACACTGCTGGTTTACACTGTTTCTCCAAGCCATCA  5473
AC210774.3_7565-13867/1-6303         ---CAACTTGGACAGTGCCCCAAAAA-CCTGTCATCCCTACTATCTTCTGTCTAGTCATACTCCCATTCACCGTTCTCAATTACTCATAAATGCCCTGCTCTTGTTTACACTGCTGGTTTACACTGCTGGTTTACACTGTTTCTCCAAGCCATCA  5473

Consensus_HERV-H/1-8613              CAGCTGATATCTCTTGGTGCTATCCCCAAACCGCCACTCTTAATTCCCTCTTAGAGTGGGTAGATGATCTTTGCTGGCAGGGCACCCTCCAATACTTCCACCCTGATGAAGTTCTATTCTTTACTTTTATACTCTCTCTTATTCTCATTCCCATT  6341
chr4_91045770-91046171/1-402         -----------------------------------------------------------------------------------------------------------------------------------------------------------    41
AC226758.3_c17895-11593/1-6303       CAGCTGAGATCTCCTGGTGCTAACCCCAGACTGCCACTCTTAACTCCCTCTTAAA----GTAAATAATCTTTGCTGGCAGGGC------------------------------------------------------------------------  5552
AC210774.3_7565-13867/1-6303         CAGCTGAGATCTCCTGGTGCTAACCCCAGACTGCCACTCTTAACTCCCTCTTAAA----GTAAATAATCTTTGCTGGCAGGGC------------------------------------------------------------------------  5552

Consensus_HERV-H/1-8613              CTTATGCCACCTTTTGCCTCTCCCCAGCTATCTCCACCACACTATCAACCTTACCCATTCTCTCCTAGCTGCTTCTAATCCCTCCTTAGTGAACAACTGCTGGCTTTGCATTTCCCTTTCTTCCAGTGCCTACATAGCTGTCCCTGCCTTACAGA  6496
chr4_91045770-91046171/1-402         -----------------------------------------------------------------------------------------------------------------------------------------------------------    41
AC226758.3_c17895-11593/1-6303       -----------------------------------------------------------------------------------------------------------------------------------------------------------  5552
AC210774.3_7565-13867/1-6303         -----------------------------------------------------------------------------------------------------------------------------------------------------------  5552

Consensus_HERV-H/1-8613              CAGACTGGGCTACATCTCCTGTCTCCTTACACCTCTGAACTTCCTTTAACAGCCCTCACCTTTACCCTCCTAAGGAACTCATTTACTTTCTAGACAGGTCCAGCAAGACTTTCCCAGACATTTCACATCAGCAAGCTGCCGCCCTCCTCCGCACT  6651
chr4_91045770-91046171/1-402         -----------------------------------------------------------------------------------------------------------------------------------------------------------    41
AC226758.3_c17895-11593/1-6303       -----------------------------------------------------------------------------------------------------------------------------------------------------------  5552
AC210774.3_7565-13867/1-6303         -----------------------------------------------------------------------------------------------------------------------------------------------------------  5552

Consensus_HERV-H/1-8613              TATTTAAAAAACCTTTCTCCTTATATTAACTCTACTCCCCCCATATTTGGACCTCTCACAACACAAACTATTATTCCTGTTGCTGCTCCTTTATGTATCTCTTGGCAAAGACCCACTGGAATTCCCCTAGGTAATATTTCACCTTCTTGATGTTC  6806
chr4_91045770-91046171/1-402         -----------------------------------------------------------------------------------------------------------------------------------------------------------    41
AC226758.3_c17895-11593/1-6303       -----------------------------------------------------------------------------------------------------------------------------------------------------------  5552
AC210774.3_7565-13867/1-6303         -----------------------------------------------------------------------------------------------------------------------------------------------------------  5552

Consensus_HERV-H/1-8613              CTTTACTCTTTATCTCCAAAGCCCAACTACACACATCACTGAAACAATTGGAGCCTTCCAGCTCCATATTACAGACAAGCCCTCTGTCAATACTGACAAACTCAAAAACATTAGCAGTAATTGTTGCTTAGGAAGACACTTACCCTGTATTTCAC  6961
chr4_91045770-91046171/1-402         -----------------------------------------------------------------------------------------------------------------------------------------------------------    41
AC226758.3_c17895-11593/1-6303       -----------------------------------------------------------------------------------------------------------------------------------------------------------  5552
AC210774.3_7565-13867/1-6303         -----------------------------------------------------------------------------------------------------------------------------------------------------------  5552

Consensus_HERV-H/1-8613              TCCATCCTTGGCTACCTTCCCCTTGCTCATCAGACTCTCCTCCCAGGCCCTCTTCTTGTTTACTTATACCCAGCCCCCAAAATAACAGTGAAAGGTTGCTCATAGATAATCAACGTTTTCTCGTACATCATGAAAATTGAACATCCTCCTCTATG  7116
chr4_91045770-91046171/1-402         -----------------------------------------------------------------------------------------------------------------------------------------------------------    41
AC226758.3_c17895-11593/1-6303       -----------------------------------------------------------------------------------------------------------------------------------------------------------  5552
AC210774.3_7565-13867/1-6303         -----------------------------------------------------------------------------------------------------------------------------------------------------------  5552

Consensus_HERV-H/1-8613              CAGTTACCCCATCAGTCCCCATTACAACCTCTGACAGCTGCCGCCCTAGCTGGATCCCTAGGAGTCTGGGTACAAGACACCCCTTTCAGCACTCCTTTTCACTTTTTTACTTTACATCTCCAGTTTTGCCTCACACAAGGTCTCTTCTTCCTCTG  7271
chr4_91045770-91046171/1-402         -----------------------------------------------------------------------------------------------------------------------------------------------------------    41
AC226758.3_c17895-11593/1-6303       -----------------------------------------------------------------------------------------------------------------------------------------------------------  5552
AC210774.3_7565-13867/1-6303         -----------------------------------------------------------------------------------------------------------------------------------------------------------  5552
```

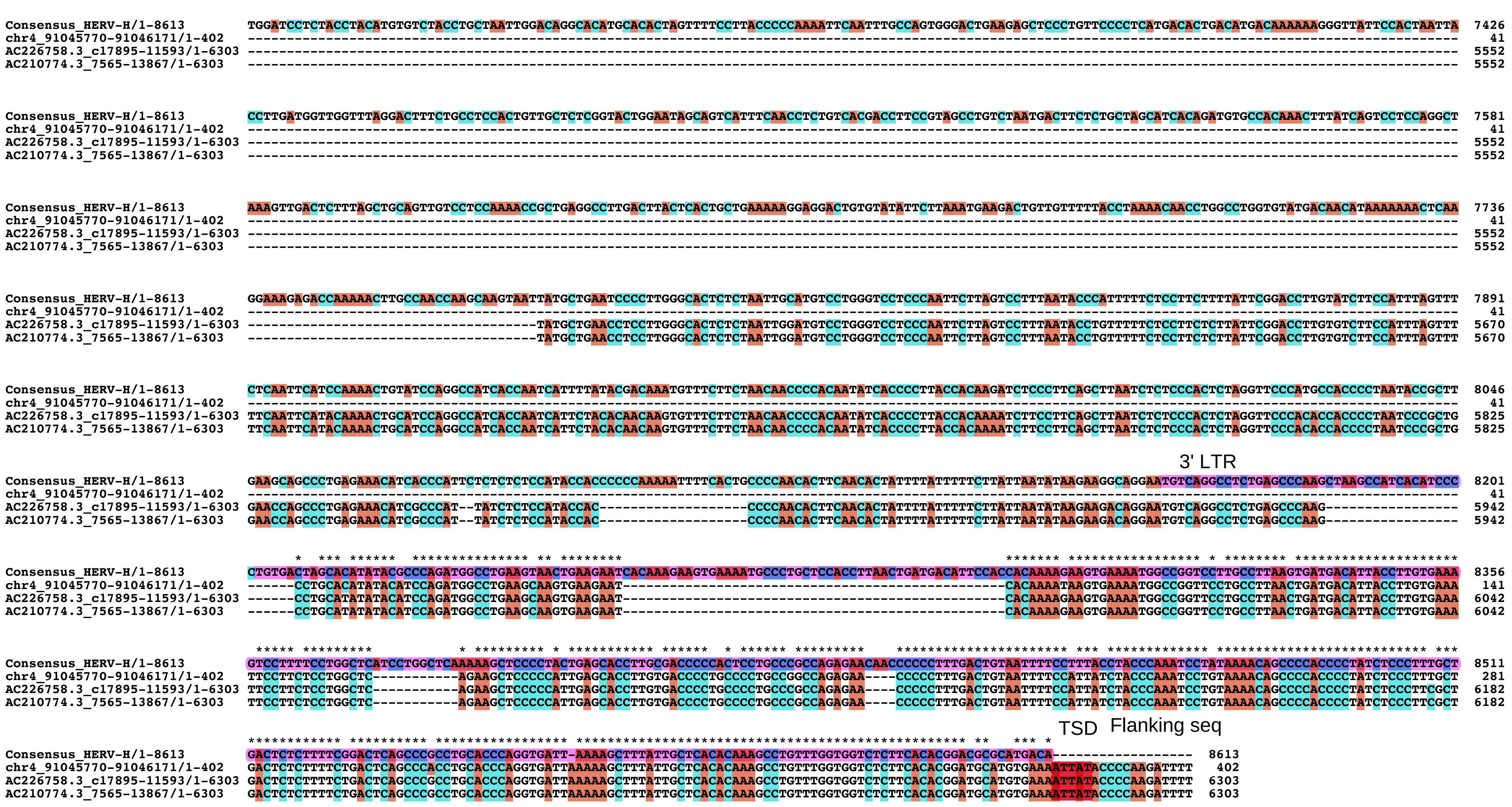

Additional file 6: Alignment of HERV-H consensus with 4q22.1_H8 solo LTR and provirus. The target site duplications (TSD) is marked in red. The 5' and 3'LTRs are highlighted in pink. The HERV-H consensus sequence is from Repbase (LTR7 and HERVH).
