# Supplementary material for: Variation in proviral content among human genomes mediated by LTR recombination
Source: Mob DNA. 2018 Dec 18;9:36. doi: 10.1186/s13100-018-0142-3 (PMC6298018; doi:10.1186/s13100-018-0142-3)
Supplement: Supplementary file 7 — Alignment of 5p15.31_H2 solo LTR and provirus with HERV-H consensus. (PDF 350 kb) [file 13100_2018_142_MOESM7_ESM.pdf]

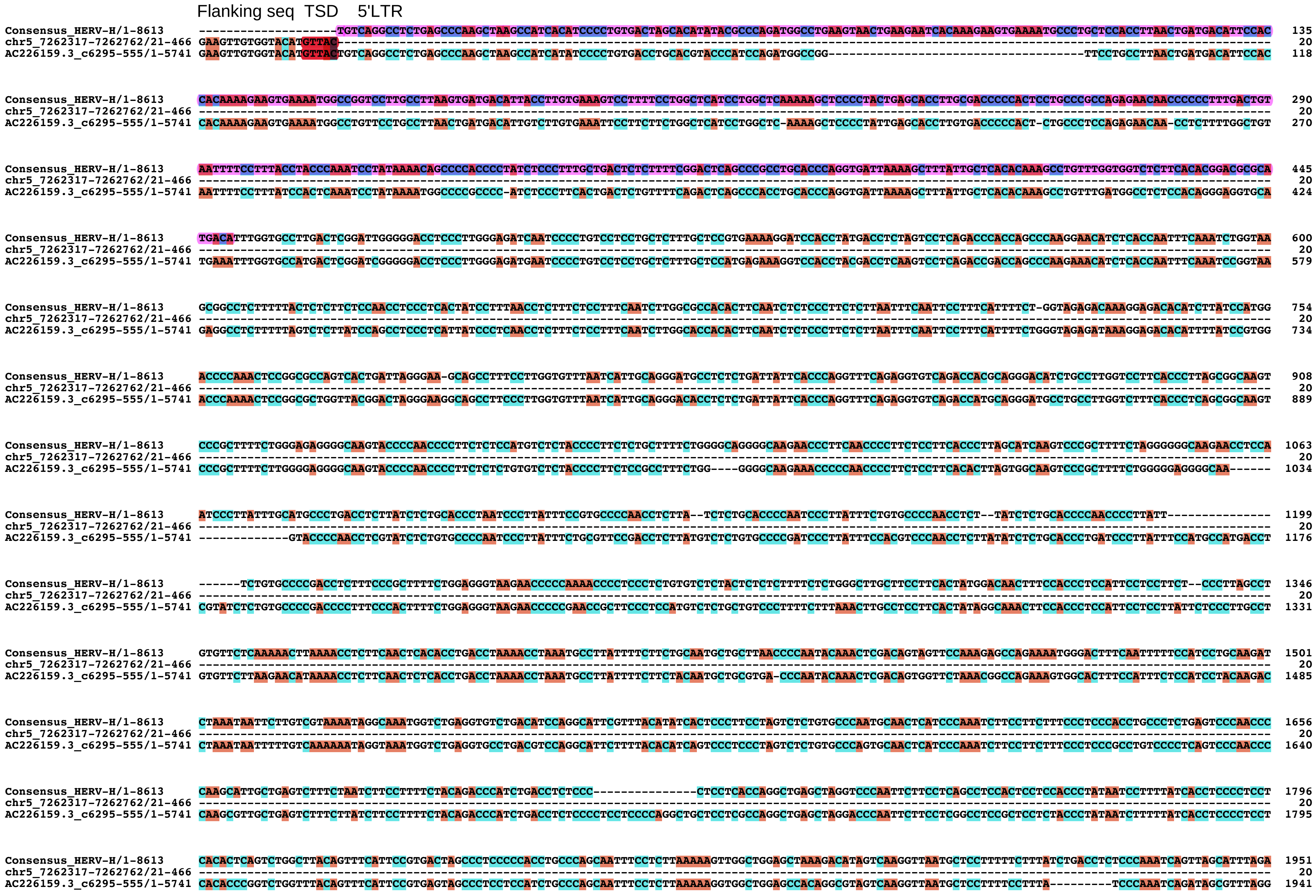

Flanking seq
TSD
5'LTR
Consensus_HERV-H/1-8613
chr5_7262317-7262762/21-466
AC226159.3_c6295-555/1-5741

```
Consensus_HERV-H/1-8613       CTCTTTTTCATCAAATACAAAAAACCCAGCCCAGTTCATGGCTCATTCGGCAGCAACCCTGAGACGCTTTACAGCCTTAGACCCTAAAAGGTCAAAAGGCCGTCTTATCCTCAATATACATTTTATTACCCAATCTGCTCCAAACATTAAATAAA 2106
chr5_7262317-7262762/21-466   ----------------------------------------------------------------------------------------------------------------------------------------------------------- 20
AC226159.3_c6295-555/1-5741   CTCTTTTTCATCAAATATGAAAAACCCAACCCAGTTCATGGCTCGTTCGGCAGCAACCCTGAGACGCTTTACAGCCCTAGACCCTAAAAGGTCAAAAGGTCGTCTTATTCTCAATATACATTTTATTACCCAATCTGCTCCCAACATTAAATAAA 2096

Consensus_HERV-H/1-8613       ACTCCAAAAATTAAATTCTGGCCCTCAAACCCCACAACAGGATTTAATTAACCTCACCTTCAAGGTGTACAA-TAATAGAAAA---------------------------AAGTTGCAATTCCTTGCCTCCACTGTGAGACAAACCCCAGACACA 2233
chr5_7262317-7262762/21-466   ----------------------------------------------------------------------------------------------------------------------------------------------------------- 20
AC226159.3_c6295-555/1-5741   ACTCCAAAAATTAAATTCTGGCCCTCAAACCCCACAACAGGACTTAATTAACCTCACCTTCAAGGTGTACAATTAATAGAGTAGAGGCAGCCAAGTAGCAACATATTTCTGAGTTGCGATTCTTTGCCTCTACTGTGAGACAAACCCCAGCTACA 2251

Consensus_HERV-H/1-8613       TCTCCAGCACACAAGAACTTCGAAATGCCTCAACCTCAGGTGCCAGGGGTTCCTCCAGAACCTTCTCCCCCAGGAGCTTGCTACAAGTGCCAGAAATCTGGCCACTGGGCCAAGGAATGCCCACAGACCAGGATTCCTCCTAAGCTGTATCCCAT 2388
chr5_7262317-7262762/21-466   ----------------------------------------------------------------------------------------------------------------------------------------------------------- 20
AC226159.3_c6295-555/1-5741   TCTCCAGCACACAAGAACTCC-AAACGCCTGAACCGCAGCTGCCAGGGGTTTCTCCAGAACCTCCTCCCCCAGGAGCTTGCTACAAGTGCCGGAAATCTGGCCACTGGGCCAAGGAATGCCCACAGCCCAAGATTCTTCCTAAGCCATGTCCCAT 2405

Consensus_HERV-H/1-8613       CTCTGTGGGACCCCACTAAAAATCAGACTGTTCAACTCACCTGGCAGCCACTTCCAGAGCCCCTGGAACTCTAGCCCAAGGCTCTCTGACTGACCCCTTCTGAGATCTTCTTGGCTTAGCAGCTGAAGACTGACACTGCCAGATCGCCTCGGAAG 2543
chr5_7262317-7262762/21-466   ----------------------------------------------------------------------------------------------------------------------------------------------------------- 20
AC226159.3_c6295-555/1-5741   CTGTGTGGGACCCCACTGAAAATTGGACTGTTCAACTCACCTGGCAGCCACTTCCAGAGGCCCTGGAACTCTGGCCCAAGGCTCTCTGACTGACTTCTCCCCAGATCTTCTTGGCTTAACAGCTGAAGACTGACACTGCCCGATTGCCTCAGAAG 2560

Consensus_HERV-H/1-8613       CCTACAGGACCATCACAGATGCTCCAGGTAACTCTCACAGTAGAGGGTAAGTCTGTCCCCTTCTTAATCAATATGGAGGCTACCCACTGCACATTACCTTCTTTTCAAGGGCCTGTTTCCTTTGCCTCCATAACTGTTGTGGGTATTGACGGCCA 2698
chr5_7262317-7262762/21-466   ----------------------------------------------------------------------------------------------------------------------------------------------------------- 20
AC226159.3_c6295-555/1-5741   CCTACAGGACCATCACAGACGCTCTAGGAAACTCTCACAGTGGAAGGTAAGTCCGTCCCCTTCTT----AATACAGAGGCTACTCACTCCACATTACCTTATTTTCAAGGGCCTGTCTCCCTTGCTTCCATAATTGTTGTGCATATTGATGGCCA 2711

Consensus_HERV-H/1-8613       GGCTTCTAAACCTCTTAAAACTCCCCAACTCTAGTACCAACTTAGACAATACTCTTTTAAGCACTCCTTTTTAGTTATCCCCACTTGCCCAGTTCCCTTATGAGGCCGAGACACTTCAACTAAATTATCTGCTTCCCTGACTATTCCTGGACTAC 2853
chr5_7262317-7262762/21-466   ----------------------------------------------------------------------------------------------------------------------------------------------------------- 20
AC226159.3_c6295-555/1-5741   GGCTTCTAAACCTCTTTAAACTCCCCAACTCTGGTGCCAACTTAGACAATACTCTTTTAAGTACTCCTTTTTAATTATCCCCACCTGCCCAGTTCCCTTATTAGGCCGAGACACTT----TAAATTATCTGCTTCCCTGACTGTTCCTAGGCTAC 2862

Consensus_HERV-H/1-8613       AGCTACATCTCATTGCTGCCCTTCTTCCCAATCCAAAGCCTCCTTTGCATCTT---CTTGTATCCCCCAACCTTAACCCACAAGTATAAGATACCTCTATTCCCTCCTTGGTGACCAATCATGCACCCCTTACCATCTCATTAAAACCTAATCAC 3005
chr5_7262317-7262762/21-466   ----------------------------------------------------------------------------------------------------------------------------------------------------------- 20
AC226159.3_c6295-555/1-5741   AGCCACACCTCATTGCCACCTTTTCCCGCCGTTTAAAGCTTCCTTCACGTCCTCCCCTTGTATCTCCCCACCTTAACCCACAAGTATAAGACACCTCTACTCCCCCCTTAGTGACCAATCATGCACCCCTTACCATCCCATTAAAACCTAATCAC 3017

Consensus_HERV-H/1-8613       TCTTACCCGGCTCAATGCCAAGATCCCATCCCACAGCATGCTTTAAAAGGATTAAAACCTGTTATCACTCGCCTGCTAGAGCATGGCCTTTTAAAGCCTATAAACTCTCCTTACAATTCCCCCATTTTACCTGTCCTAGAACCAGACAAGCCTTA 3160
chr5_7262317-7262762/21-466   ----------------------------------------------------------------------------------------------------------------------------------------------------------- 20
AC226159.3_c6295-555/1-5741   TCTTACCCCGCTCAATGCCAATATCCCACCCCACAGCATGCTTTGAAAGGATTAAAGCCTGTTATCGCTCACCTGTTACAGTATGGCCTTTTAAAGCCTATAAACTCTTCTTACCATT--CCCATTTTACCTGTCCTAAAACCAGACAAGGCTTA 3170

Consensus_HERV-H/1-8613       CAG----GTTCAGGATCTGTGTCTTATCAATGAAATTGTTTTCCCTATCCACCCTGTGGTGCTGAACCCATATACTCTCCTATCCTCAATACCTCCCTCTACAACCCATTATTCTGTTCTAGATCTCAAACATGCTTTCTTTACTATCCCTTTAC 3311
chr5_7262317-7262762/21-466   ----------------------------------------------------------------------------------------------------------------------------------------------------------- 20
AC226159.3_c6295-555/1-5741   CAGGTTAGTTCAGAATCTACGCCTTATCAACCAAATTGTTTTGCCTATCCACCCCGTGTTGCCAAAACCATATACTCTCTTATCCTCAATACCTCCCTCTACAACCCATTATTCTGTTCTGGATCTCAAACATGCTTTCTTTA--------TTGC 3317

Consensus_HERV-H/1-8613       ACCCTTCAACCCAGCCTCTCTTCGTTTTCACCTGGACTGACCCTGACACCCATCAGTCCCAGCAGCTTACCTGGGCTGTAATGCTGCAAGGTTTCAGGGGCAGCCCTTATTATTTCAGCCAAGCTCTTTCTCATGATTTACTTTCTTTCCACCCC 3466
chr5_7262317-7262762/21-466   ----------------------------------------------------------------------------------------------------------------------------------------------------------- 20
AC226159.3_c6295-555/1-5741   ACCCTTCATCCCAGCCTCTCTTCCCTTTCACTTAGACTGACCCTGACACCCATCAGGCTCAGCAAATTACCTGGGCTGTATTGCCGCAAGGCTTTGCAGATAGCCCCCATTACTTCAGTC----------------------------------- 3437

Consensus_HERV-H/1-8613       TCCACTTCTCACCTTATTCAATATATTGGTGATGTTCTTCTTTGTAGCCCCTCCTTTGAATCTTCTCAACAAGACACACTTCTGCTCCTTCAGCATTTATTCTCCAAAGGATATCCCCCTCCAAAGCTCAAATGTCTTCTCCATCCGTTACCTAC 3621
chr5_7262317-7262762/21-466   ----------------------------------------------------------------------------------------------------------------------------------------------------------- 20
AC226159.3_c6295-555/1-5741   ---------------------------------------------------------------------------------------------------------------------------AAGCCCAAATTTCATCCTCATCTGTTACCTAT 3469

Consensus_HERV-H/1-8613       CTTGGCATAATTCTTCATAAAAACACACGTGCCCTCCCTGCTGATAGTGTCTGACTGATCTCTCAAACCCCAACCCCTTCTACAAAACAACAACTCTTTTCCATCCTAGGCATGGTTGGATACTTTCGTGTTAGGATACCTGGTTTTGCCATCCT 3776
chr5_7262317-7262762/21-466   ----------------------------------------------------------------------------------------------------------------------------------------------------------- 20
AC226159.3_c6295-555/1-5741   CTCGGCATAATTC-TCATAAAAACACACGTGCTCTCCCTGCTGATCGTGTCCATTTCATCTCCCAAACCTCAATCCCT--TACAAAACAACAACTCCTTTCCTTCCTAGGCATGGT--------------------------------------- 3582

Consensus_HERV-H/1-8613       AACAAAACCATTATATAAACTCACAAAAGGAAACCTAGTTGACCCCATAGATCCTAAATCGTTTCCCCACTCCTCTTTCCATTCCTTGAAGACAGCTTTAGAGACTGTCTCCACTCTAGCTCTCCCTGACTCATCCCAACACTTTTCATTACACA 3931
chr5_7262317-7262762/21-466   ----------------------------------------------------------------------------------------------------------------------------------------------------------- 20
AC226159.3_c6295-555/1-5741   ----------------------------------------------------------------------------------------------------------------------------------------------------------- 3582
```

```
Consensus_HERV-H/1-8613          CAGCTGAAGTGCAGGGCTGTGCAGTCAGAATTCTTACACAAGGACCGGGATCGCATCCTGTAGCCTTTTTGTCCAAACAACTTGACCTTACTGTTTTAGGCTGGCCATCATGTCTCCATGCAGCGTCTGCTGCCACCCTAATACTTTTAGAGGCC  4086
chr5_7262317-7262762/21-466      -----------------------------------------------------------------------------------------------------------------------------------------------------------    20
AC226159.3_c6295-555/1-5741      ----------------CAGTGTGGTCAGAATTCTTACACAAGAGCCAGGGCCGTACCCTGTAGCCTTTCTGTCCAAACAACTTGACCTTACTTTTTTAGCCTAGCCCTCATGTCTGCAAGTAGCAGCTGCCGCTGCTTTAATACTTTTAGAGGCC  3721

Consensus_HERV-H/1-8613          CTCAAAATCACAAACTATGCTCAACTCATTCTCTACAGCTCTCATAATTTCCAAAATCTATTTTCTTCCTCACACCTGACACATATACTTTCTGCTCCCCGGCTCCTTCAGATATACTCACTCCATTTATTCTCCCACAATTACCATTATTCCTG  4241
chr5_7262317-7262762/21-466      -----------------------------------------------------------------------------------------------------------------------------------------------------------    20
AC226159.3_c6295-555/1-5741      CTAAAAATCACAAACTACGCTCAACTCA--ATCTACATTTCTCATAACTTCCAAAATCCATTTTCTTCCTCATACCTGATGCATATACTTTCTGCTCCCCGGCTCCTTCAGCTGTAATCACTCTTTCTTAAGTCCCACAATTACCATTGTTCCTG  3874

Consensus_HERV-H/1-8613          GCCTGGACTTCAATCCGGCCTCCCACATTATTCTGGATACCATACCTGACCCTCATGACTGCATCTCTCTGATCCACCTGACGTTCACCCCATTTCCCCACATTTCCTTCTGCCCTGTTTCTCACCCTGATCACACTTGGTTTATTGATGGCAGT  4396
chr5_7262317-7262762/21-466      -----------------------------------------------------------------------------------------------------------------------------------------------------------    20
AC226159.3_c6295-555/1-5741      GCCCAGACTTCAATCCGGCCTCCTACATTATTCTGGATACCACACCTGACCCTCATGACTGTCTCTCTCTGATCCACCTGACATTCACCCCATTTCCCCATATTTCCTTCTTTCCTGTTCCTCACCCTGATCACACTTGATTTATTGATGTCAGT  4029

Consensus_HERV-H/1-8613          TCCACCAGGCCTAATCGCCACTCACCAGCAAAGGCAGGATATGCTATGAACTAGTTGCCTTAATTCAAGCCCTCACTCTTGCAAAAGGACTACGTGTCAATATCTATACTGATTCTAAATATGCCTTTCATATTCTGCACCACCATGCGGTCATA  4551
chr5_7262317-7262762/21-466      -----------------------------------------------------------------------------------------------------------------------------------------------------------    20
AC226159.3_c6295-555/1-5741      TCCACTAGGCCTAATCGCCACACACCAGCAAAGGCAGGCTATGCTATAG----------------------------------------------------------------------------------------------------------  4078

Consensus_HERV-H/1-8613          TGGGCTGAAAGAGGTTTCCTCACTACACAAGTGTCCTCCATCATTAATGCCTCTTTAAGAAAACTCTGCTCAAGGCTGCTTTACTTCCAAAGGAAGCTGGGGTCATTCACTGCAAGGGGCATCAAAAGACTTCAGATCCCATTGCTCTAGGCAAT  4706
chr5_7262317-7262762/21-466      -----------------------------------------------------------------------------------------------------------------------------------------------------------    20
AC226159.3_c6295-555/1-5741      -----------------------------------------------------------------------------------------------------------------------------------------------------------  4078

Consensus_HERV-H/1-8613          GCTTATGCTGATAAGGTGGCTAGACAAGCAGCTAGCTCTCCAACTTTTGTCCCTCATGGCCAGTTTTTCTCCTTCACATCCGTCACTCCCACCTACTCCACAGCTGAAACTTCCACCTATCAAGCTCTTCCCCCGCAAGGTAAATGGTTCTTAGA  4861
chr5_7262317-7262762/21-466      -----------------------------------------------------------------------------------------------------------------------------------------------------------    20
AC226159.3_c6295-555/1-5741      -----------------------------------------------------------------------------------------------------------------------------------------------------------  4078

Consensus_HERV-H/1-8613          CCAAGGAAAATATCTCCTTCCAGCCTCACAGGCCCATTCTATTCTGTCGTCATTTCATAACCTTTTCCATGTAGGTTACAAGCCACTAGCCTGTCTCTTAGGACCTCTCATTTCCTTTCCATCATGGAAATCTATCCTCAAGGAGATCACTTCTC  5016
chr5_7262317-7262762/21-466      -----------------------------------------------------------------------------------------------------------------------------------------------------------    20
AC226159.3_c6295-555/1-5741      ----------------------------------------------------------------------------TACAAGCCACTAGCCCGCCTCTTAGAACCTCTTATTTCCTTTCCATCGTGGAAATCTAACCTCAAGGAAGTAACCTCTC  4157

Consensus_HERV-H/1-8613          AGTGTTCCATCTGCTATTCTGCTACCCCTCAGGGATTGTTCAGGCCTCCTCCCTTTCCTACACATAAAGCTCGGGGATTTGCCCCTGCCCAGGACTGGCAAATTGACTTTACTC-ACATGCCTCGGGTCAGAAAACTAAAATATCTCTTAGTCTG  5170
chr5_7262317-7262762/21-466      -----------------------------------------------------------------------------------------------------------------------------------------------------------    20
AC226159.3_c6295-555/1-5741      AGTGTTCCATCTGCTATTCTACTACTCCTCAGGGATTATTCAGGCCCCCTCCCTTCCATACACATCAAGCTTGAAGATTTGCCCCCACCCAGGACTGGCAAATTAGCTTTACTCAACATGCCCTGAGTCAGATAGCTAAAATACCTCTTAGTCTA  4312

Consensus_HERV-H/1-8613          GGTAGACACTTTCACTGGGTGGGTAGAGGCCTTTCCCATAGAGTCTGAGAAGGCCACCGCGGTCATTTCTTCCCTTCTGTCAGACATAATTCCTTGGTTTGGCCTTCCCTTCTCTATACAGTCTGATAACGGACCAGCCTTTACTAGTTAAATCA  5325
chr5_7262317-7262762/21-466      -----------------------------------------------------------------------------------------------------------------------------------------------------------    20
AC226159.3_c6295-555/1-5741      GGTAGACACTTCCACTGGATAGGTACAGG-------------GTCTGAGAAGGCCACCACAGTCATTTCTTCCCTTCTGTCAGACATAATTCCTCAGTTTAGCCTTCCCACCTCTATACAGTCTGATAACAGACCAGCCTTCATTAGTCAAATCA  4454

Consensus_HERV-H/1-8613          CCCAAGCAGTTTCTCAGGCTCTTGGTATTCAGTGGAACCTTCATATCCCTTAACATCCTCAATCTTCAGGAAAGGTAAAACCGACTAATGGTCTTTTAAAGACACACCTCACCAAGCTCAGCCTCCAACTTAAAAAGGATTGGACAGTACTTTTA  5480
chr5_7262317-7262762/21-466      -----------------------------------------------------------------------------------------------------------------------------------------------------------    20
AC226159.3_c6295-555/1-5741      GCCAAGCAGTTTTTCAGGCTCTTAGTATTCAGTGAAACCTTTATATCCCTTACGGTCCTCCGTCTTCAGGAAAAGTAGAACGGACTAAAGGTCTTTTAAAAACACACCTCCCCAAGCTCAGCCACCAACTTAAAAAGGACTGGACAATACTTTTA  4609

Consensus_HERV-H/1-8613          CCTCTCGCCCTTCTCAGAATTAGAACCTGTCCTCGAGATGCTACAGGGTACAGTCCATTTGAACTTTTATATGGACGCACTTTCTTGCTTGGTCCCAACCTCATCCCAGACACCAGCCCTCTAGGCGACTATCTTCCAGTCCTCCAACAGGCTAG  5635
chr5_7262317-7262762/21-466      -----------------------------------------------------------------------------------------------------------------------------------------------------------    20
AC226159.3_c6295-555/1-5741      CCACTTTCCCTTCTCAGAAGTCAGACCTGCCCTCAGAATGCTACAGGATACAGCCCATTTGAGCTCCTGTATAGATGCTCCTTTTTATTAGGCCCCAGTCTCATTCCAGACACCAGAC-------------------------------------  4727

Consensus_HERV-H/1-8613          GCAGGAAAGTCACCAGGCTGCTAATCTTCTCTTGCCTACTCCAGATCCCCAGCCATATGAAGACACTCTAGCTGGACGATCAGTTCTTGTTAAGAATCTGACCCCTCAAACTCTACAACCTCGATGGACTGGACCCTACTTAGTCATCTATAGTA  5790
chr5_7262317-7262762/21-466      -----------------------------------------------------------------------------------------------------------------------------------------------------------    20
AC226159.3_c6295-555/1-5741      -----------------------------------------------------------------------------------------------------------------------------------------------------------  4727

Consensus_HERV-H/1-8613          CCCTGACTGCCGTCCGCCTGCAGGATCCTCCCCACTGGGTTCACCATTCCAGAATAAAGCTGTGTCCATTGGACAGCCAGCCTAATCCCTCCTCTTCCTCCTGGAAGTCGCAATTACTCTCCCCTACTTCCCTTAAACTCACTCGTATTTCTGAA  5945
chr5_7262317-7262762/21-466      -----------------------------------------------------------------------------------------------------------------------------------------------------------    20
AC226159.3_c6295-555/1-5741      -----------------------------------------------------------------------------------------------------------------------------------------------------------  4727
```

```
Consensus_HERV-H/1-8613          GAACAGTAATAACCCTTATGAGCCTAATACATCCCTTCATTCTATTAGGTCTGTTTGTCCTTACCCTACTTTTTGCAACAGGGCTTTATGAACTCACCCCCACCACTTAGGCTGAGCCCAAAAAATCTTGTCATCCCTACTATTTTCTGTCCAGT 6100
chr5_7262317-7262762/21-466      ----------------------------------------------------------------------------------------------------------------------------------------------------------- 20
AC226159.3_c6295-555/1-5741      ------------------------------------------------------------------------------------------------------CAACTTAGACTGTGCCCCAAAAAAATTGTCATCCCTACTATCTTCTGTCTAGT 4780

Consensus_HERV-H/1-8613          CATACTCCTATTCTCTGCTCTCAACTACTTATAAATGCCGTACTCTTGTTTACACTGCTGGTTTACACTGTTTCTTCAAGCCATCACAGCTGATATCTCTTGGTGCTATCCCCAAACCGCCACTCTTAATTCCCTCTTAGAGTGGGTAGATGATC 6255
chr5_7262317-7262762/21-466      ----------------------------------------------------------------------------------------------------------------------------------------------------------- 20
AC226159.3_c6295-555/1-5741      CATACTCCTATTCACCATTCTCAACTACTCATACATGCCCTGCTCTTGTTTACACTGCCGGTTTACACTGTTTTTCCAAGCCATCACAGCTGATATCTCCTGGTGCTATCCCCAAACTGCCACTCTTAA----CTCTTGAAGTAAATAAATAATC 4931

Consensus_HERV-H/1-8613          TTTGCTGGCAGGGCACCCTCCAATACTTCCACCCTGATGAAGTTCTATTCTTTACTTTTATACTCTCTCTTATTCTCATTCCCATTCTTATGCCACCTTTTGCCTCTCCCCAGCTATCTCCACCACACTATCAACCTTACCCATTCTCTCCTAGC 6410
chr5_7262317-7262762/21-466      ----------------------------------------------------------------------------------------------------------------------------------------------------------- 20
AC226159.3_c6295-555/1-5741      TTTGCTGAAAGGAC--------------------------------------------------------------------------------------------------------------------------------------------- 4945

Consensus_HERV-H/1-8613          TGCTTCTAATCCCTCCTTAGTGAACAACTGCTGGCTTTGCATTTCCCTTTCTTCCAGTGCCTACATAGCTGTCCCTGCCTTACAGACAGACTGGGCTACATCTCCTGTCTCCTTACACCTCTGAACTTCCTTTAACAGCCCTCACCTTTACCCTC 6565
chr5_7262317-7262762/21-466      ----------------------------------------------------------------------------------------------------------------------------------------------------------- 20
AC226159.3_c6295-555/1-5741      ----------------------------------------------------------------------------------------------------------------------------------------------------------- 4945

Consensus_HERV-H/1-8613          CTAAGGAACTCATTTACTTTCTAGACAGGTCCAGCAAGACTTTCCCAGACATTTCACATCAGCAAGCTGCCGCCCTCCTCCGCACTTATTTAAAAAACCTTTCTCCTTATATTAACTCTACTCCCCCCATATTTGGACCTCTCACAACACAAACT 6720
chr5_7262317-7262762/21-466      ----------------------------------------------------------------------------------------------------------------------------------------------------------- 20
AC226159.3_c6295-555/1-5741      ----------------------------------------------------------------------------------------------------------------------------------------------------------- 4945

Consensus_HERV-H/1-8613          ATTATTCCTGTTGCTGCTCCTTTATGTATCTCTTGGCAAAGACCCACTGGAATTCCCCTAGGTAATATTTCACCTTCTTGATGTTCCTTTACTCTTTATCTCCAAAGCCCAACTACACACATCACTGAAACAATTGGAGCCTTCCAGCTCCATAT 6875
chr5_7262317-7262762/21-466      ----------------------------------------------------------------------------------------------------------------------------------------------------------- 20
AC226159.3_c6295-555/1-5741      ----------------------------------------------------------------------------------------------------------------------------------------------------------- 4945

Consensus_HERV-H/1-8613          TACAGACAAGCCCTCTGTCAATACTGACAAACTCAAAAACATTAGCAGTAATTGTTGCTTAGGAAGACACTTACCCTGTATTTCACTCCATCCTTGGCTACCTTCCCCTTGCTCATCAGACTCTCCTCCCAGGCCCTCTTCTTGTTTACTTATAC 7030
chr5_7262317-7262762/21-466      ----------------------------------------------------------------------------------------------------------------------------------------------------------- 20
AC226159.3_c6295-555/1-5741      ----------------------------------------------------------------------------------------------------------------------------------------------------------- 4945

Consensus_HERV-H/1-8613          CCAGCCCCCAAAATAACAGTGAAAGGTTGCTCATAGATAATCAACGTTTTCTCGTACATCATGAAAATTGAACATCCTCCTCTATGCAGTTACCCCATCAGTCCCCATTACAACCTCTGACAGCTGCCGCCCTAGCTGGATCCCTAGGAGTCTGG 7185
chr5_7262317-7262762/21-466      ----------------------------------------------------------------------------------------------------------------------------------------------------------- 20
AC226159.3_c6295-555/1-5741      ----------------------------------------------------------------------------------------------------------------------------------------------------------- 4945

Consensus_HERV-H/1-8613          GTACAAGACACCCCTTTCAGCACTCCTTTTCACTTTTTTACTTTACATCTCCAGTTTTGCCTCACACAAGGTCTCTTCTTCCTCTGTGGATCCTCTACCTACATGTGTCTACCTGCTAATTGGACAGGCACATGCACACTAGTTTTCCTTACCCC 7340
chr5_7262317-7262762/21-466      ----------------------------------------------------------------------------------------------------------------------------------------------------------- 20
AC226159.3_c6295-555/1-5741      ----------------------------------------------------------------------------------------------------------------------------------------------------------- 4945

Consensus_HERV-H/1-8613          CAAAATTCAATTTGCCAGTGGGACTGAAGAGCTCCCTGTTCCCCTCATGACACTGACATGACAAAAAAGGGTTATTCCACTAATTACCTTGATGGTTGGTTTAGGACTTTCTGCCTCCACTGTTGCTCTCGGTACTGGAATAGCAGTCATTTCAA 7495
chr5_7262317-7262762/21-466      ----------------------------------------------------------------------------------------------------------------------------------------------------------- 20
AC226159.3_c6295-555/1-5741      ----------------------------------------------------------------------------------------------------------------------------------------------------------- 4945

Consensus_HERV-H/1-8613          CCTCTGTCACGACCTTCCGTAGCCTGTCTAATGACTTCTCTGCTAGCATCACAGATGTGCCACAAACTTTATCAGTCCTCCAGGCTAAAGTTGACTCTTTAGCTGCAGTTGTCCTCCAAAACCGCTGAGGCCTTGACTTACTCACTGCTGAAAAA 7650
chr5_7262317-7262762/21-466      ----------------------------------------------------------------------------------------------------------------------------------------------------------- 20
AC226159.3_c6295-555/1-5741      ----------------------------------------------------------------------------------------------------------------------------------------------------------- 4945

Consensus_HERV-H/1-8613          GGAGGACTGTGTATATTCTTAAATGAAGACTGTTGTTTTTACCTAAAACAACCTGGCCTGGTGTATGACAACATAAAAAAACTCAAGGAAAGAGACCAAAAACTTGCCAACCAAGCAAGTAATTATGCTGAATCCCCTTGGGCACTCTCTAATTG 7805
chr5_7262317-7262762/21-466      ----------------------------------------------------------------------------------------------------------------------------------------------------------- 20
AC226159.3_c6295-555/1-5741      ---------------------------------------------------------------------------------------------------------------------------TATGCTGAATCTCCTTAGGCA--CTCTAATTA 4975

Consensus_HERV-H/1-8613          CATGTCCTGGGTCCTCCCAATTCTTAGTCCTTTAATACCCATTTTTCTCCTTCTTTTATTCGGACCTTGTATCTTCCATTTAGTTTCTCAATTCATCCAAAACTGTATCCAGGCCATCACCAATCATTTTATACGACAAATGTTTCTTCTAACAA 7960
chr5_7262317-7262762/21-466      ----------------------------------------------------------------------------------------------------------------------------------------------------------- 20
AC226159.3_c6295-555/1-5741      GATGTCCTGGGTCCTCCCAAATCTTAGACCTTTTATACCTGTTTTTCTCCTTCTGTTA---------------TTCCATTTAGTTTTTCAATTCATGCAAAACCATATCCAGGCCATCACCAATCATTCTAAATGACAAATGTCTCTTCTAACAG 5115
```

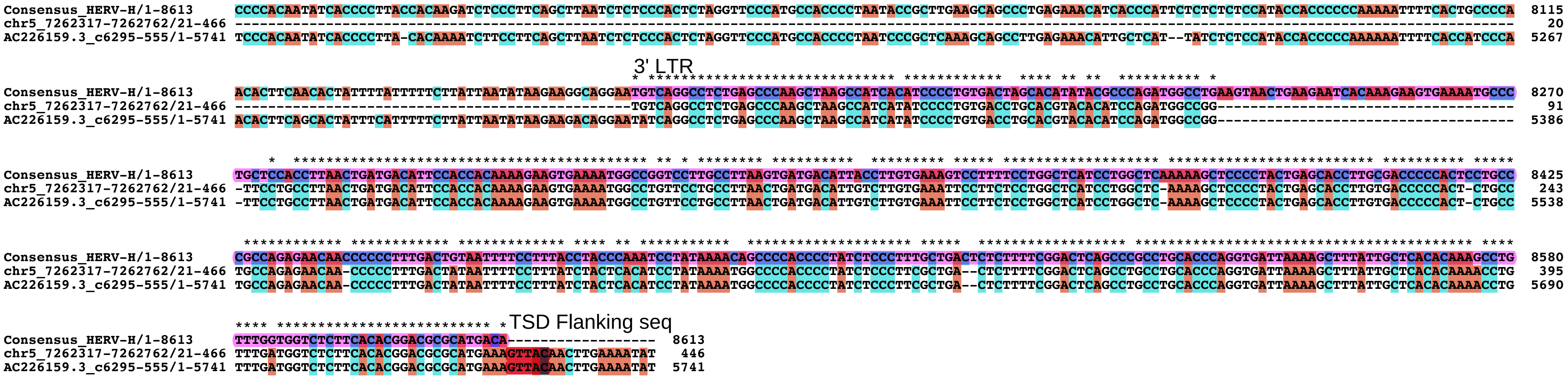

Additional file 7: Alignment of HERV-H consensus with 5p15.31_H2 solo LTR and provirus. The target site duplications (TSD) are marked in red. The 5' and 3'LTRs are highlighted in pink. The HERV-H consensus sequence is from Repbase (LTR7 and HERVH).
