# Supplementary material for: Variation in proviral content among human genomes mediated by LTR recombination
Source: Mob DNA. 2018 Dec 18;9:36. doi: 10.1186/s13100-018-0142-3 (PMC6298018; doi:10.1186/s13100-018-0142-3)
Supplement: Supplementary file 8 — IGV screenshot of dimorphic HERV-H (2q34_H4) locus. (PDF 36 kb) [file 13100_2018_142_MOESM8_ESM.pdf]

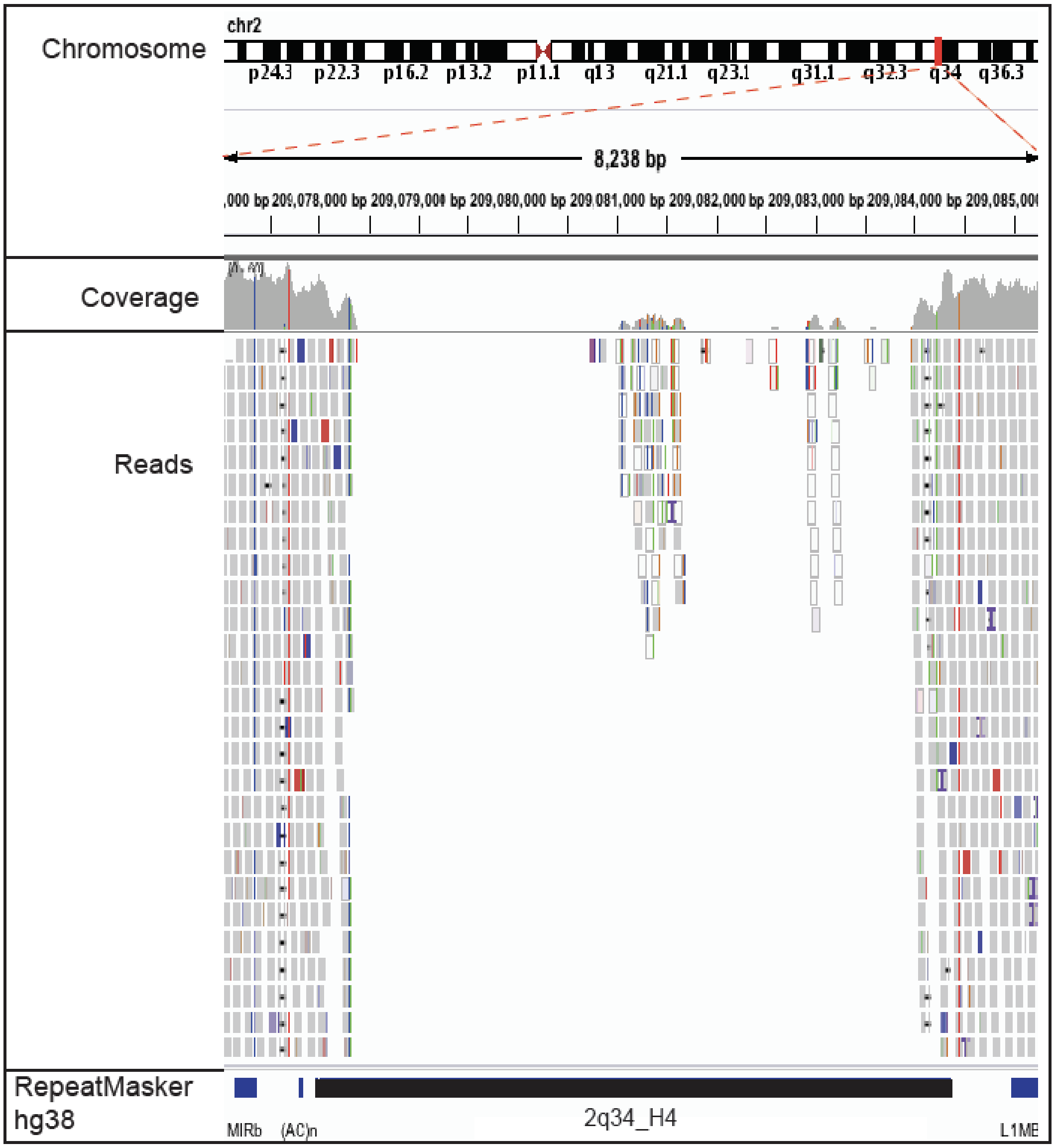

Additional file 8: IGV screenshot of dimorphic HERV-H (2q34_H4) locus. The top panel shows the position on the chromosome and hg38 coordinates. The Coverage track shows the number of reads that covers each base at the locus. The numbers within brackets [0-60] shows the range of reads covering the visualized region. The Reads track shows a sample of the reads mapped to that locus. The RepeatMasker track shows the position of transposable elements and repeats identified in that chromosomal interval. Here, nearly a complete lack of uniquely mapping reads to the internal region suggest the presence of two solo LTR alleles.
