# Supplementary material for: Variation in proviral content among human genomes mediated by LTR recombination
Source: Mob DNA. 2018 Dec 18;9:36. doi: 10.1186/s13100-018-0142-3 (PMC6298018; doi:10.1186/s13100-018-0142-3)
Supplement: Supplementary file 9 — HERV candidates identified as provirus to solo LTR variants using findsoloLTR pipeline. (PDF 723 kb) [file 13100_2018_142_MOESM9_ESM.pdf]

Additional file 9. HERV candidates identified as provirus to solo LTR variants using *findsoloLTR* pipeline

HERV-name	Hg38 coordinates	#Individuals	Read depth (%)	Avg. mappability for the provirus	DGV Accession	Presence of Refseq/ LINC RNA/ envORF	Expression type in hiPSC	Previously identified
1p31.1_K3	chr1:75377086-75383458	NA	5-47	0.055	essv3655324	*env		(28)
1q22_K2	chr1:155626666-155635845	NA	5-15	0.07		TCONS_l2_00002667 TCONS_l2_00001553 *env		This study
11q22.1_K1	chr11:101695063-101704528	NA	0-32	0.066	essv4365330	*env		(28)
11q23.3_K1	chr11:118721015-118730174	2	46-50	0.177		*env		This study
12q14.1_K1	chr12:58327459-58336915	NA	3-45	0.072	nssv585653	*env		(28)
3q13.2_K2	chr3:113024277-113033435	NA	2-30	0.077	esv1096242	*env		(38)
3q27.2_K1	chr3:185562548-185571727	NA	5-43	0.084		*env		(43)
5p13.3_K2	chr5:30486653-30496098	77	6-50	0.145	nsv499389			This study (PCR verified)
5q33.3_K1	chr5:156657706-156666885	NA	4-27	0.062	$essv12110787	*env		(43)
6q14.1_K1	chr6:77716945-77726366	NA	5-43	0.075	nsv499600	TCONS_l2_00024299 *env		(28)
7p22.1_K1	chr7:4582426-4591897	NA	5-20	0.058	*nssv619986 (+240bp)	*env		(28,38)
8p23.1_K7	chr8:7497875-7507337	NA	0-49	0.064	$nsv1075518	*env		This study (likely a pre-insertion☽)
11q13.2_H5	chr11:68633778-68639439	11	6-49	0.198	esv2672143		MA	This study
2q34_H4	chr2:209078020-209084376	95	4-49	0.173	nsv498954		MA	This study (PCR verified)

2p14_H2	chr2:64252414-64257646	55	6-48	0.125	esv2670157	NR_120420.1 TCONS_l2_00013678 TCONS_l2_00013679	HA	This study
3q26.31_H8	chr3:175827485-175836221	2	3-45	0.271				This study
4q31.21_H2	chr4:145698823-145703503	2	6-36	0.189			HA	This study
Xq21.33_H1	chrX:94698820-94704626	2	6	0.126			HA	This study
Xq22.1_H4	chrX:99158722-99165186	3	6-7	0.216				This study
13q21.32_H1	chr13:66141332-66147036	15	9-49	0.145	esv3632499		MA	This study
1q31.1_H1	chr1:185948279-185954216	4	29-39	0.075			MA	This study
1q32.2_H3	chr1:210111090-210116207	4	45-49	0.374	esv3588715			This study
1q42.2_H3	chr1:232118115-232123945	23	40-50	0.09			HA	This study
1p32.3_H6	chr1:54897904-54903584	1	47.34	0.207	esv3586106			This study
1p31.3_H3	chr1:65643207-65648034	1	49.88	0.142			MA	This study
10q21.1_H3	chr10:53492723-53504727	3	45-48	0.097			MA	This study
10q21.1_H4	chr10:54166676-54172501	21	41-50	0.111			MA	This study
10p14_H2	chr10:6797083-6802953	79	40-50	0.135	*nsv1130992 (+245bp)	TCONS_00018113 TCONS_00018114 TCONS_00017700	MA	This study
11q24.3_H2	chr11:130753498-130759137	2	49	0.151	essv14312658		MA	This study
11p14.3_H1	chr11:23183934-23189744	6	32-46	0.157	esv3547446		MA	This study
12p13.2_H1	chr12:10332976-10338643	5	32-49	0.137			MA	This study
12p12.1_H2	chr12:25163213-25169508	1	42	0.254	essv14414920			This study

13q14.11_H2	chr13:42868004-42873158	48	44-49	0.11		NR_126389.1	HA	This study
13q21.1_H1	chr13:55578228-55584087	150	21-49	0.117	nssv3984585		HA	This study
13q22.3_H1	chr13:77933223-77939379	1	43	0.247	essv14801756			This study
13q31.1_H1	chr13:79274082-79279821	1	49.21	0.14				This study
14q21.3_H1	chr14:47104197-47108765	40	42-50	0.098			HA	This study
15q26.2_H4	chr15:96871837-96877667	1	40	0.135	*nssv2999175 (+866bp)		HA	This study
19q13.42_H1	chr19:53831404-53837348	1	35	0.136			HA	This study
2q24.1_H4	chr2:156720084-156725844	2	46-49	0.167			MA	This study
2q34_H1	chr2:208215925-208221935	1	44	0.211	*essv10832987 (+94bp)			This study
2q36.1_H5	chr2:224296633-224302363	5	46-49	0.17	essv10865359			This study
2p12_H2	chr2:75213731-75219537	4	47-50	0.147	nsv953151		MA	This study
2p12_H4	chr2:76573625-76579704	1	49	0.142			MA	This study
2p12_H5	chr2:77088247-77094030	5	43-49	0.12			HA	This study
20p12.1_H4	chr20:13357880-13362689	7	42-49	0.122			HA	This study
21p11.2_H7	chr21:9847516-9850363	1	47	-				This study
3q22.3_H2	chr3:137595601-137601190	1	43	0.226	esv2741215			This study
3q27.3_H2	chr3:187175504-187181262	2	49	0.091			HA	This study
3q28_H10	chr3:192071109-192076858	1	49	0.191	*essv11273010 (+320bp)			This study
3p24.3_H2	chr3:20967541-20973231	5	20-50	0.201			MA	This study

3p14.3_H1	chr3:54634484-54640204	2	39-49	0.132	essv11015497	ESRG	HA	This study (PCR verified)
3p12.3_H6	chr3:78581212-78588919	218	39-50	0.135	*nsv954897 (+261bp)		MA	This study
3p25.3_H3	chr3:8177031-8182716	1	35	0.164	*esv3561425 (+217bp)		MA	This study
4q26_H1	chr4:115975700-115981225	1	48	0.147	*nssv2996771 (+719bp)	TCONS_l2_00021950		This study
4q27_H1	chr4:119993664-119999666	3	41-46	0.114	*nsv949813 (+418bp, +279bp)		HA	This study
4q31.1_H1	chr4:138927836-138933543	1	34	0.181		TCONS_00007856 TCONS_00007857		This study
4q32.3_H5	chr4:166716125-166722054	1	48	0.13	nssv2996802		MA	This study
4q22.2_H1	chr4:94084647-94089891	2	47-50	0.116			MA	This study
5q21.2_H3	chr5:104063635-104070481	1	49	0.21	*essv12004941 (+472bp)		MA	This study
5p14.3_H3	chr5:18668843-18674598	14	43-50	0.147	*nssv2997714 (+351bp, +293bp)		HA	This study
5q13.3_H2	chr5:76357256-76363071	2	42-47	0.13			MA	This study
6q22.1_H2	chr6:115031793-115037619	6	30-45	0.117	*essv12509186 (+326bp)		MA	This study
6q22.31_H9	chr6:125701848-125707763	5	45-50	0.11	*nssv2997515 (-757bp)	TCONS_00011367	HA	This study
6q23.2_H3	chr6:131338800-131344564	4	41-50	0.159	essv12552764		MA	This study
6q23.2_H7	chr6:131901550-131907417	24	39-50	0.129	*nssv2997521 (+489bp)	TCONS_l2_00025237 TCONS_l2_00025238 TCONS_l2_00025239	HA	This study
6p22.3_H1	chr6:16259012-16264893	5	43-50	0.139	*essv12200615 (+72bp)		MA	This study
6p22.3_H3	chr6:18754144-18759870	2	40-43	0.169	essv12211245	TCONS_00012100	MA	This study

6p12.2_H1	chr6:51938241-51944426	2	48	0.206	essv12349521			This study
6q15_H2	chr6:90670205-90675442	1	49.5	0.086			MA	This study
6q16.1_H1	chr6:93830156-93835749	20	41-50	0.096	essv12465823		HA	This study
8q23.3_H6	chr8:115212253-115217978	1	40	0.116			MA	This study
9q33.1_H3	chr9:115839178-115844929	2	38-45	0.094		TCONS_00016140	MA	This study
9q21.13_H2	chr9:74085583-74091436	1	48	0.125	*essv5998236 (-1172bp, -1691bp)		HA	This study
9q21.33_H8	chr9:87410694-87416706	1	49	0.114			HA	This study
9q22.2_H1	chr9:90412695-90418349	43	41-49	0.113		TCONS_00015842	MA	This study
Xq23_H9	chrX:114466672-114472531	7	46-49	0.116			HA	This study
Xq23_H10	chrX:114601272-114607242	65	41-50	0.09			HA	This study
Xp22.32_H2	chrX:4891614-4897331	1	11	0.114			MA	This study

Footnotes: #Individuals identified: NA: Not applicable: we did not calculate the number of individuals when the mappability is too low. DGV variant accession: Accessions marked with asterisks (*) have either one or two boundaries outside the LTR or inside in the internal sequence. The presence of a single number within brackets next to accession shows that one breakpoint of DGV variant is not within the LTR. The presence of two numbers within the brackets shows that both breakpoints of the DGV variant are not within the LTRs. The numbers within the bracket next to accession indicate the how far the breakpoint is from the boundary of the provirus. (+) indicates break point is outside of LTR and (-) indicates break point is internal to the element from the boundary. Accessions marked with ($) are indicative of a pre-integration allele as both breakpoints precisely lie at the outer boundaries of LTR. There could be multiple variants present in DGV; only one representative variant accession is given here. Previously identified: ☽ insertion polymorphism (pre-integration allele for the provirus) has been identified in (29). Presence of Refseq / LINC RNA / envORF: *env encodes a complete envelope gene of > 400 amino acids according to Heidmann et al. (92). Expression type in hiPSCells: MA: moderately active, HA: Highly active, Expression type of full length HERVH identified in hiPSC lines and single cells derived from three individual hESC clones (63).
