# Supplementary material for: Variation in proviral content among human genomes mediated by LTR recombination
Source: Mob DNA. 2018 Dec 18;9:36. doi: 10.1186/s13100-018-0142-3 (PMC6298018; doi:10.1186/s13100-018-0142-3)
Supplement: Supplementary file 10 — List of primer sequences used for amplifying solo LTR and provirus alleles shown in Fig. 3. (PDF 124 kb) [file 13100_2018_142_MOESM10_ESM.pdf]

Additional file 10. List of primer sequences used for amplifying solo LTR and provirus alleles shown in Figure 3

HERV_allele	Forward Primer (5' to 3')	Reverse Primer (5' to 3')	AT(^{0}C)/ ET (sec)
18q21.1_W2_solo	GAGTGCACCTGTTAGGCTATTT	TCTCCCTCACCTGCTTCTATC	52/60
18q21.1_W2_env	GAGTGCACCTGTTAGGCTATTT	GCCCATGCTCCAATGTTAATG	51/60
18q21.1_W2_gag	GGACAGGTGCCTGGTAATTT	TCTCCCTCACCTGCTTCTAT	52.8/60
4q22.1_H8_solo	ACACTTAGAAGACCTCCATCTTAAT	TTGCCTGCATACACAATTCTTC	50.8/55
4q22.1_H8_env	CTTCAGCTTAATCTCTCCCACTC	GACATGGAGCACCTGGAAA	51.7/50
5p15.31_H2_env	CTTCAGCTTAATCTCTCCCACTC	CCCTTTATTTCTAGTAAGGCTTGTTG	51.6/51
2q34_H4_solo	GCAATAGTGATCTCGGGTCAAA	CTTGTTCCAATGACGACCTGTA	51.8/70
2q34_H4_env	AGACACCAGACCGACTTAGA	CTCCCTGACCACGTGATTATT	51.7/90
3p14.3_H1_solo	CCCTCACTTAGCTTCGTATTCTC	GGTCTTCCCTGACTATGGTTTC	51.7/45
3p14.3_H1_gag	CTTCCCTCACTTAGCTTCGTATT	GAGATGTTTCTTGGGCTGGT	51.7/45
5p13.3_K2_solo*	CTGGGATGCAAGTCTTGTTTA	TGTATTCTGCCATTCACATTTC	60.2/135
5p13.3_K2_env*	TGCAGGTGTAACCAACAG	TGTATTCTGCCATTCACATTTC	60.2/135

Footnotes: AT- Annealing temperature, ET- Extension time, PCR conditions for *Go Taq* PCR Master mix- Promega: an initial denaturation temperature of 95ºC for 2 minutes, followed by 36 cycles of (i) denaturation at 95ºC for 30 sec, (ii) annealing at 50-53ºC for 30 sec, (iii) extension of 72ºC for a minute per kilo base (see ET above) and a final extension of 72ºC for 5 minutes. *PCR conditions for *Platinum SuperFI* PCR Master Mix- Thermo Fisher Scientific: an initial denaturation temperature of 98ºC for 30 seconds, followed by 35 cycles of (i) denaturation at 98ºC for 10 sec, (ii) annealing at 60.2ºC for 10 sec, (iii) extension of 72ºC for 2 minutes 15 sec and a final extension of 72ºC for 5 minutes
